# Ca^2+^ binding to synapsin I regulates resting Ca^2+^ and recovery from synaptic depression in nerve terminals

**DOI:** 10.1007/s00018-022-04631-5

**Published:** 2022-11-21

**Authors:** Matteo Moschetta, Tiziana Ravasenga, Antonio De Fusco, Luca Maragliano, Davide Aprile, Marta Orlando, Silvio Sacchetti, Silvia Casagrande, Gabriele Lignani, Anna Fassio, Pietro Baldelli, Fabio Benfenati

**Affiliations:** 1 Center for Synaptic Neuroscience and Technology, Istituto Italiano Di Tecnologia, Largo Rosanna Benzi 10, 16132 Genoa, Italy; 2 Department of Experimental Medicine, University of Genova, Viale Benedetto XV, 3, 16132 Genoa, Italy; 3 IRCCS, Ospedale Policlinico San Martino, Largo Rosanna Benzi 10, 16132 Genoa, Italy; 4 Department of Life and Environmental Sciences, Polytechnic University of Marche, Via Brecce Bianche, 60131 Ancona, Italy; 5 Present Address: Charitè Universitätsmedizin Berlin, Freie Universität Berlin and Humboldt-Universität Zu Berlin, Berlin Institute of Health, NeuroCure Cluster of Excellence, Berlin, Germany; 6 Present Address: Queens Square Institute of Neurology, University College London, London, UK; 7 Present Address: High-Definition Disease Modelling Lab, Campus IFOM-IEO, Milan, Italy

**Keywords:** Synaptic transmission, Synaptic vesicles, Spontaneous release, Short-term plasticity, Excitatory and inhibitory neurons

## Abstract

Synapsin I (SynI) is a synaptic vesicle (SV)-associated phosphoprotein that modulates neurotransmission by controlling SV trafficking. The SynI C-domain contains a highly conserved ATP binding site mediating SynI oligomerization and SV clustering and an adjacent main Ca^2+^ binding site, whose physiological role is unexplored. Molecular dynamics simulations revealed that the E373K point mutation irreversibly deletes Ca^2+^ binding to SynI, still allowing ATP binding, but inducing a destabilization of the SynI oligomerization interface. Here, we analyzed the effects of this mutation on neurotransmitter release and short-term plasticity in excitatory and inhibitory synapses from primary hippocampal neurons. Patch-clamp recordings showed an increase in the frequency of miniature excitatory postsynaptic currents (EPSCs) that was totally occluded by exogenous Ca^2+^ chelators and associated with a constitutive increase in resting terminal Ca^2+^ concentrations. Evoked EPSC amplitude was also reduced, due to a decreased readily releasable pool (RRP) size. Moreover, in both excitatory and inhibitory synapses, we observed a marked impaired recovery from synaptic depression, associated with impaired RRP refilling and depletion of the recycling pool of SVs. Our study identifies SynI as a novel Ca^2+^ buffer in excitatory terminals. Blocking Ca^2+^ binding to SynI results in higher constitutive Ca^2+^ levels that increase the probability of spontaneous release and disperse SVs. This causes a decreased size of the RRP and an impaired recovery from depression due to the failure of SV reclustering after sustained high-frequency stimulation. The results indicate a physiological role of Ca^2+^ binding to SynI in the regulation of SV clustering and trafficking in nerve terminals.

## Introduction

A cohort of proteins operates synaptic vesicle (SV) exocytosis at nerve terminals and fine-tune the efficiency of neurotransmitter release. Three main functional SV pools exist: a readily releasable pool (RRP) of SVs docked/primed at the active zone, a recycling pool (RecP) of SVs that refill the RRP depleted upon the activity, and a resting pool (RestP) of SVs that cannot be released by electrical activity and represent a functional reserve [2, 18, 68]. The Synapsins (SynI/II/III) constitute an abundant family of SV-associated phosphoproteins that play important roles in synaptogenesis and in the regulation of clustering and mobilization of SVs in presynaptic terminals [11, 20, 28, 50, 56]. SynI and SynII, the major isoforms in mature neurons, have both redundant and distinct functions in synaptic transmission. While both contribute to the formation and maintenance of the RecP by promoting SV clustering [7, 8, 40, 58], they have complementary roles in synchronous and asynchronous release [29, 53, 54]. Moreover, SynI is at the convergence of multiple signaling pathways and is phosphorylated during neural activity [43]. Phosphorylation of SynI by Ca^2+^/calmodulin (CaM)-dependent and cAMP-dependent kinases inactivates SV clustering by SynI, releasing SVs toward the RRP [12, 13], while phosphorylation/dephosphorylation of SynI by Cdk5 and calcineurin, respectively, regulates the transitions of SVs from the RestP to the RecP [83]. All Syn isoforms display a highly conserved ATP binding site in the central C-domain [23]. The binding of ATP was initially reported to be differentially regulated by Ca^2+^ ions in the three Syn isoforms, being stimulated, unaffected, or inhibited in Syns I, II, and III, respectively [39] and proposed to increase SynI oligomerization [10]. Afterward, molecular dynamics (MD) simulations revealed that ATP binding is mediated by a conformational transition of a flexible loop (multifunctional loop, MFL) that opens to render the ATP site accessible to the ligand [62]. This conformational change is not affected by the absence of Ca^2+^ and increases the association of SynI with SVs and SV clustering [62]. These findings raise questions on the functional role of Ca^2+^ binding to SynI. In the SynI C- domain crystal structure, Ca^2+^ is coordinated by the pyrophosphate moiety of ATP and two glutamate residues (Glu373 and Glu386; [23]) and its presence shifts the ATP-induced SynI dimerization to tetramerization [62]. Since Ca^2+^ recruitment is coordinated by Glu373 and is abolished by Glu-Lys substitution without affecting the conformation of the ATP binding pocket [39, 62], we dissected the functional role of Ca^2+^ binding to SynI by re-introducing either wild-type SynI (SynI^WT^) or the SynI^E373K^ mutant in SynI knockout (KO) neurons. MD simulations revealed that ATP and Ca^2+^ binding to SynI can occur independently, although they facilitate each other, and that the E373K mutation affects the oligomerization interface of SynI. We found that the deletion of Ca^2+^ binding to SynI increases the resting levels of intraterminal Ca^2+^ and the probability of spontaneous SV exocytosis in excitatory neurons. Moreover, expression of the SynI^E373K^ mutant decreases SV density and size of the RRP, resulting in a smaller amplitude of evoked excitatory currents. Such effects are not present in inhibitory synapses, indicating that, the Ca^2+^-buffering activity of SynI is dispensable in inhibitory neurons. In both excitatory and inhibitory synapses, however, deletion of Ca^2+^ binding to SynI leads to SV depletion and markedly impairs recovery from synaptic depression. The results indicate SynI as a new Ca^2+^-buffer for excitatory neurons and indicate that Ca^2+^ binding to SynI has important roles in regulating SV trafficking across functional SV pools.

## Materials and methods

### MD simulations

MD simulations of solvated SynI^WT^ and SynI^E373K^ C-domains were previously analyzed [62] using the program NAMD [66] and the CHARMM27 force field with the CMAP correction for protein backbone energetics [51]. Simulations lasted 150 ns and were performed at 300 K. All parameters and set-ups of the simulations are described in Orlando et al. [62]. The various simulated systems were built by immersing the protein monomer in a box of TIP3P model water molecules [42] and counter-ions to neutralize the total charge, for a total of 63,000 atoms in all cases. ATP or Ca^2+^ was removed where necessary by deleting the corresponding atoms and the mutations were introduced by replacing the side chains of the involved residues. In all simulations, periodic boundary conditions were used to replicate the system and remove box surface effects [30]. Short-range nonbonded interactions were cutoff at 12 Å, whereas long-range electrostatic interactions were computed using the particle mesh Ewald method [19]. Chemical bonds connecting hydrogen atoms to heavy atoms were kept fixed using SHAKE [71]. The integration time step was 1 fs to ensure the stability of the molecular dynamics algorithm. A proper size for the simulation box corresponding to a pressure of 1 atm was obtained by simulating the system in the constant pressure and temperature ensemble using the Nosé́-Hoover Langevin piston method for 0.5 ns [52]. The simulation ensemble was then switched to a constant volume and temperature (NVT) ensemble for the rest of the simulation by keeping the temperature stationary at 300 K using Langevin dynamics. Protein atomic coordinates were obtained from the crystal structure of rat SynI C-domain complexed with ATP and Ca^2+^ (PDB code 1pk8; [10]. A 200 ns-long simulation of the wild-type (WT) SynI C-domain without ATP and with one Ca^2+^ at the binding site was performed here with the same parameters and set-ups of the WT and E373K trajectories. Calcium ion pulling simulations were performed starting from conformations extracted from the WT ATP/no-ATP Ca^2+^ trajectories and using a harmonic restraint on the distance between the ion and the Ca^2+^ atoms of residues V372, E373, E386, and V387, with a target linearly increasing from the starting value (about 7 Å) to 40 Å in 10 ns. Five simulations were performed for ATP/Ca^2+^ SynI and five for no-ATP/Ca^2+^ SynI, always using the same force constant *k* = 0.8 kcal/(mol*Å^2^).

### Experimental animals

Syn I KO mice were generated by homologous recombination and extensively backcrossed on the C57BL/6J background for > 10 generations [48]. Mice were housed under constant temperature (22 ± 1 °C) and humidity (50%) conditions with a 12 h light/dark cycle and were provided with food and water ad libitum. All experiments were carried out in accordance with the guidelines established by the European Community Council (Directive 2010/63/EU of September 22, 2010) and were approved by the Italian Ministry of Health (authorization no. 605/2021-PR).

### Low-density and autaptic cultures of SynI KO hippocampal neurons

Pregnant females were sacrificed by inhalation of CO_2_, and embryonic day 17–18 (E17–18) embryos were removed immediately by cesarean section. Hippocampi were digested in 0.125% trypsin for 15 min, mechanically dissociated with a fire-polished Pasteur pipette, and finally plated at low density (40 cells/mm^2^). Autaptic neurons were prepared as described previously [6]. Briefly, dissociated neurons were plated at very low density (20 cells/mm^2^) on microdots (40–400 µm in diameter) obtained by spraying a mixture of poly-l-lysine (0.1 mg/mL) on dishes which had been pretreated with 0.15% agarose. All hippocampal cultures were maintained in a culture medium consisting of Neurobasal, 2% B-27, 1% Glutamax, and 1% penicillin/streptomycin in a humidified 5% CO_2_ atmosphere at 37 °C.

### Virus production and neuron transduction

Sequences containing mCherry-tagged Syn^WT^ and SynI^E373K^ were cloned into pLenti6.2/V5-Dest plasmids (Invitrogen). As a control for viral transduction, an empty EGFP-expressing lentiviral construct pCCL-sin-PPT-prom-EGFP-WPRE was used (referred to in the manuscript as SynI^KO^). The production of VSV-pseudo-typed third-generation lentiviruses was performed as previously described [21]. Viral titers of about 1.0 × 10^9^ TU/mL were obtained for all vectors. For all the experiments, hippocampal neurons were infected at 6–7 DIV at 10-multiplicity of infection. After 24 h from the infection, half of the medium was replaced with fresh medium. The transduction efficiency was assessed by counting the number of mCherry-positive cells with respect to the number of Hoechst-positive nuclei using ImageJ.

### Patch-clamp recordings from dissociated and autaptic cultured hippocampal neurons

Cultured hippocampal neurons obtained from Syn I KO mice embryos were recorded at 12–15 DIV. Patch pipettes, prepared from thin borosilicate glass, were pulled and fire-polished to a final resistance of 4–5 MΩ when filled with a standard internal solution. Voltage-clamp recordings were performed at a holding potential of − 70 mV and acquired at 10–20 kHz sample frequency. All experiments were performed at room temperature (22–24 °C). Data acquisition was performed using the PatchMaster program (HEKA Elektronic). For all the experiments, cells were maintained in an extracellular standard solution (Tyrode) containing (in mM): 140 NaCl, 2 CaCl_2_, 1 MgCl_2_, 4 KCl, 10 glucose, and 10 HEPES (pH 7.3 with NaOH). The internal solution (K-gluconate) was composed of (in mM) 126 K gluconate, 4 NaCl, 1 MgSO_4_, 0.02 CaCl_2_, 0.1 BAPTA, 15 glucose, 5 HEPES, 3 ATP, and 0.1 GTP (pH 7.3 with KOH). Evoked excitatory postsynaptic currents (eEPSCs) were recorded from autaptic neurons, maintained in a Tyrode external solution supplemented with: d-(–)-2-amino-5-phospho-nopentanoic acid (D-AP5; 50 μM) and bicuculline (BIC, 30 μM) to block *N*-methyl-d-aspartate (NMDA) and GABA_A_ receptors, respectively. To selectively block AMPA receptor desensitization experiments were run in the presence of cyclothiazide (CTZ; 60 µM). The autaptic neurons under study were voltage clamped at a holding potential (*V*_h_) of −70 mV. Unclamped action potentials (APs) evoking EPSCs were activated by a brief depolarization of the cell body to + 40 mV for 0.5 ms. Evoked inhibitory postsynaptic currents (eIPSCs) were recorded in low-density hippocampal neurons perfused with Tyrode solution supplemented with D-AP5 (50 μM), CNQX (10 μM), and CGP58845 (5 μM) to block NMDA, non-NMDA, and GABA_B_ receptors, respectively. eIPSCs were evoked by extracellular electrical stimulation, positioning the stimulating glass microelectrode, filled with the external Tyrode solution in the proximity of a putative presynaptic neuron and applying increasing current to generate a postsynaptic response in the postsynaptic voltage-clamped neuron. Extracellular stimulation was performed by administering test pulses of 0.5 ms, generally ranging between 10 and 60 pA. In particular, the stimulation intensity was progressively increased to evoke minimal eIPSCs and such value was further augmented by 50%, in order to avoid “failures” during tetanic high frequency stimulation. The size of the readily releasable pool of synchronous release (RRP_syn_) and the probability release (*P*_r_) was calculated using the cumulative amplitude analysis during 2–2.5 s tetanic stimulation at 40 Hz [73]. Data points in the linear range of the curves (between 0.5 and 1 s) were fitted by linear regression and retrogradely extrapolated to time 0. The intercept with the *Y*-axis gave the RRP and the ratio between the amplitude of the first ePSC (*I*_1_) and RRP yielded the Pr. To study the response to paired-pulse protocols, we applied two consecutive stimuli at increasing interpulse intervals (20–1000 ms). The paired-pulse ratio (PPR; *I*_2_/*I*_1_) was calculated by dividing the amplitude of the second response (*I*_2_) by the amplitude of the first one (*I*_1_). Miniature postsynaptic currents (mPSCs) were recorded in the voltage-clamp configuration in the presence of tetrodotoxin (TTX; 300 nM) in the extracellular solution to block the generation and propagation of spontaneous action potentials. The amplitude and frequency of mPSCs were calculated using a peak detector function using appropriate threshold amplitude and area. The frequency, amplitude, and kinetics of miniature PSCs were analyzed using the MiniAnalysis program (Synaptosoft, Inc.) and the Prism software (GraphPad Software, Inc.). All reagents were obtained by Tocris, otherwise specified.

### Immunofluorescence

Primary hippocampal neurons were washed once in phosphate-buffered saline (PBS) and fixed with 4% paraformaldehyde (PFA) for 15 min at room temperature. Cells were blocked with blocking buffer [5% goat serum (Vector Labs), 0.5% Triton X-100] in PBS for 1 h at room temperature. Samples were sequentially incubated with primary antibodies overnight at 4 °C, followed by incubation with fluorochrome-conjugated secondary antibodies (Invitrogen) for 2 h at room temperature. After several washes in PBS, coverslips were mounted using Mowiol (Invitrogen). For quantification of the number of inhibitory and excitatory synapses, cultured neurons were stained for vGAT (Synaptic Systems; 1:500 dilution) and vGLUT1 (Synaptic Systems; 1:200 dilution). For the analysis of synaptic density, samples were visualized using a 63 × objective in a fluorescence microscope (Leica). Several z-stacks (18–35 for each image; step size, 0.35 μm) were acquired. Image analysis was performed with ImageJ by superimposing stacks of each color channel. The density of puncta was calculated as the number of vGLUT1- or vGAT-positive puncta per 30 μm of proximal dendrite length. Manders’ colocalization coefficient was calculated using the ImageJ JACoP plugin and expressed as the percentage of mCherry-SynI signal overlapping with either vGAT or vGLUT1 immunoreactivity.

### Live imaging experiments

#### Presynaptic Ca^2+^ imaging with SynGCaMP6f

Imaging was performed in hippocampal primary cultures at room temperature (22–24 °C) in Tyrode solution. Cultures were coinfected with SynGCaMP6f (#40755 Addgene; virus provided by the Viral Core Facility of Charité—Universitätsmedizin Berlin) and either SynI^WT^ or SynI^E373K^ viral vectors in a 1:1 ratio 6–7 days before the experiments and measured at 14–15 DIV. Coverslips were mounted in a Chamlide stimulation chamber (Live Cell Instrument, Seoul, Korea) on the stage of an Olympus IX-71 inverted microscope fitted with a UplanSapo 60X1.35 NA oil-immersion objective (Olympus) and an Orca-ER IEEE1394 CCD camera (Hamamatsu Photonics, Hamamatsu City, Japan, resolution 1344 × 1024). A 460–495 nm excitation/510–550 nm emission filter set was used for SynGCaMP6f. Time lapses were acquired at binning 2 with a 1 Hz frequency (200-ms exposure), for the whole experimental time. APs were evoked by passing 10 V, 1 ms current pulses from a custom-made stimulation box via platinum electrodes at increasing frequencies (0.5–100 Hz) for 2 s. Stimulation, image acquisition, and shuttering were all under the coordinated control of the WinWCP software. Images were analyzed in ImageJ and analyzed with Prism software (GraphPad Software, Inc.). Regions of interest (ROIs) were positioned on all boutons that colocalized with mCherry-positive puncta and fluorescent signals were normalized to the background. The experimental setting was designed as follows: cells were imaged for 15 s of baseline, after which a 2-s train of APs at increasing frequencies was administered and fluorescence was recorded until 30 s. For the basal recordings, the fluorescent SynGCaMP6f signals, recorded for 15 s under non-stimulated conditions, were normalized to the local background signal. For activity-dependent recordings, ROIs were positioned on all boutons responding to 20 APs for SynGCaMP6f. Evoked signals were quantified by normalizing to the local background noise and the non-stimulated (basal) signal.

#### Calcium imaging with Fura-2 AM

Primary hippocampal neurons (7 DIV) were transduced with either SynI^WT^ or SynI^E373K^ fused to mCherry and imaging was performed at 14/15 DIV. Cells were loaded with 1 μg/mL cell-permeable Fura-2 AM (#F1221, ThermoFisher) in the culture medium and maintained for 30 min in the incubator. Cells were then washed with culture medium and incubated for 30 min to allow hydrolysis of the esterified groups. Coverslips with cells were mounted in the imaging chamber and loaded with 0.5 mL of recording buffer (150 mM NaCl, 3 mM KCl, 2 mM CaCl_2_, 1 mM MgCl_2_, 10 mM glucose, and 10 mM HEPES pH 7.4). Fura-2 loaded cultures were observed with an IX-81 motorized inverted epifluorescence microscope (Olympus, Tokyo, Japan) using a UplanSapo 63 × 1.35 NA oil-immersion objective (Olympus). Regions of interest (ROIs) were positioned on all boutons that colocalized with mCherry-positive puncta. Samples were excited at 340 and 380 nm by an MT20 Hg–Xe lamp (Olympus). Excitation light was separated from the emitted light using a 395 nm dichroic mirror. Images of fluorescence emission > 510 nm were acquired continuously for 300 s using a Hamamatsu Orca-ER IEEE1394 CCD camera (Hamamatsu Photonics, Hamamatsu City, Japan). The camera operated on 2 × 2-pixel binning mode, and the imaging system was controlled by an integrating imaging software package (Cell^∧^R; Olympus). Ca^2+^ concentration was calculated as in Grynkiewicz et al. [36], using the following equation:

$$[{\text{Ca}}^{2 + } ] = K_{{\text{d}}} \cdot \frac{{R - R_{\min } }}{{R_{\max } - R}} \cdot \frac{{F_{\max }^{380} }}{{F_{\min }^{380} }}$$

where *R* is the measured 340/380 nm ratio; *R*_min_ and *R*_max_ are the ratios in the absence of Ca^2+^ or when Fura-2 is saturated by Ca^2+^ and were determined by incubating cells in 0 Ca^2+^/5 mM EGTA or treating cells with 1 μM ionomycin in recording buffer containing increasing concentrations of Ca^2+^ (from 1 nM to 10 mM). *F*^380^_max_ and *F*^380^_min_ are the fluorescence intensities of 380 nm excitation at 0 Ca^2+^ and Ca^2+^ saturation, respectively. The calibration was performed for each cell preparation by loading cells with Fura-2, then measuring the 340/380 nm ratios in Ca^2+^ buffer mixtures that covered the [Ca^2+^] range of interest (*r*^2^ = 0.883 ± 0.048; mean ± SEM). To obtain the calibration curve, values were plotted and the *x*-intercept representing the log of the *K*_d_ in the Grynkiewicz equation was calculated by linear regression. Recorded images were analyzed by ImageJ and Prism6 softwares.

#### Imaging of SV cycling with synaptopHluorin (sypHy)

Primary hippocampal neurons (10 DIV) were transduced with sypHy-GFP and either SynI^WT^ or SynI^E373K^ fused to mCherry. At 18–21 DIV live imaging recordings coupled with electric field stimulations were performed in Quick Exchange Platform (Warner Instruments) by applying 1-ms current pulses through platinum–iridium electrodes using an AM2100 stimulator (AM-Systems). Blocking of glutamate receptors in neuronal cultures was achieved by adding CNQX and APV (10 and 50 µM respectively) to the Tyrode solution. The experimental setting was designed as follows: (i) cells were imaged for 10 s (baseline); (ii) a train of 20 APs @ 100 Hz was applied and fluorescence recorded for further 90 s, a protocol repeated three times; (iii) at the end, a larger stimulus (600 AP @ 20 Hz) was applied and fluorescence recorded for 120 s; (iv) after 80 s of resting to avoid bleaching, another round of three 20 APs @ 100 Hz stimulations was applied; (v) at the end of the experimental protocol, cells were perfused with 50 mM NH_4_Cl-Tyrode to calculate the total fluorescence at responsive synaptic terminals (*F*_max_). Fluorescence intensity at synaptic boutons was measured by manually applying circular regions of interests (ROIs) of 1.7 µm diameter at the center of each responsive synapse expressing either SynI^WT^ or SynI^E373K^. The increase in the fluorescence signal (Δ*F*) upon the 20 APs @ 100 Hz stimulation normalized to the basal fluorescence (*F*_0_, calculated as the average of the first 5 s before the stimulus application after background subtraction) is a measure of the RRP (Δ*F*/*F*_0_). The endocytosis rate (τ Endo) was calculated by fitting the post-stimulus decay with the single-exponential function Δ*F*/*F*_0_ (*t*) = (Δ*F*/*F*_0_)_∞_ (*t* = ∞) + Δ*F*/*F*_0_ (*t* = 0) ⋅ e^−*t*/τ^. The normalized means of three peaks before (train 1) and after (train 2) the long-lasting stimulation were evaluated to calculate recovery. Three independent cell preparations were performed for each experimental condition and an average of 32 synaptic boutons per field were analyzed.

### Electron microscopy

Cultured hippocampal neurons derived from SynI KO mice were infected at 7 DIV with either SynI^WT^ or SynI^E373K^ and fixed at 14 DIV with 1.2% glutaraldehyde in 66 mM sodium cacodylate buffer, post-fixed in 1% OsO_4_, 1.5% K_4_Fe(CN)_6_, 0.1 M sodium cacodylate, *en bloc* stained with 1% uranyl acetate, dehydrated and flat-embedded in epoxy resin (Epon 812, TAAB). After baking for 48 h, the glass coverslips were removed from the Epon block by thermal shock and neurons were identified by means of a stereomicroscope. Embedded neurons were excised from the block and mounted on a cured Epon block for sectioning using an EM UC6 ultramicrotome (Leica Microsystems). Ultrathin sections (60–70 nm thick) were collected on mesh copper grids (Electron Microscopy Sciences) and observed with a JEM-1011 electron microscope (Jeol) operating at 100 kV using an ORIUS SC1000 CCD camera (Gatan, Pleasanton). For each experimental condition, at least 30 images of synapses were acquired at 10,000 × magnification (sampled area per experimental condition: 36 μm^2^). SV density and docked SV linear density were determined using ImageJ software. The analysis of synaptic ultrastructure at the end of the train stimulation protocol (30 s at 20 Hz) or after recovery (120 s at 0.1 Hz) was performed by quickly fixing the samples with glutaraldehyde at 37 °C. Under these conditions, we estimated a complete fixation of synapses within 1–3 s from fixative addition [47].

### Statistical analysis

Data are expressed as box plots or means ± sem (in case of normal distribution) for *n* = sample size. Normal distribution was assessed using D’Agostino and Pearson’s normality test. The box plot elements are the following: center line, median (Q2); cross symbol, mean; box limits, 25th (Q1)–75th (Q3) percentiles; whisker length is determined by the outermost data points within threefold the interquartile range (Q3–Q1). To compare two sample groups, the Student’s *t*-test and the Mann–Whitney *U*-test were used for normally and non-normally distributed data, respectively. To compare more than two normally distributed sample groups, one- or two-way ANOVA followed by multiple comparison tests (Bonferroni’s, Tukey’s, or Dunnett’s tests) were used. To compare more than two sample groups that were not normally distributed, Kruskal–Wallis’s ANOVA followed by the Dunn’s multiple comparison test was used. The significance level was preset to *p* < 0.05 for all tests. Statistical analysis was carried out using OriginPro-9 (OriginLab Corp.) and Prism (GraphPad Software, Inc.).

## Results

### The presence of ATP strengthens binding of Ca^2+^ to the main ATP/Ca^2+^ binding site in the C-domain of SynI

**Fig. 1:**
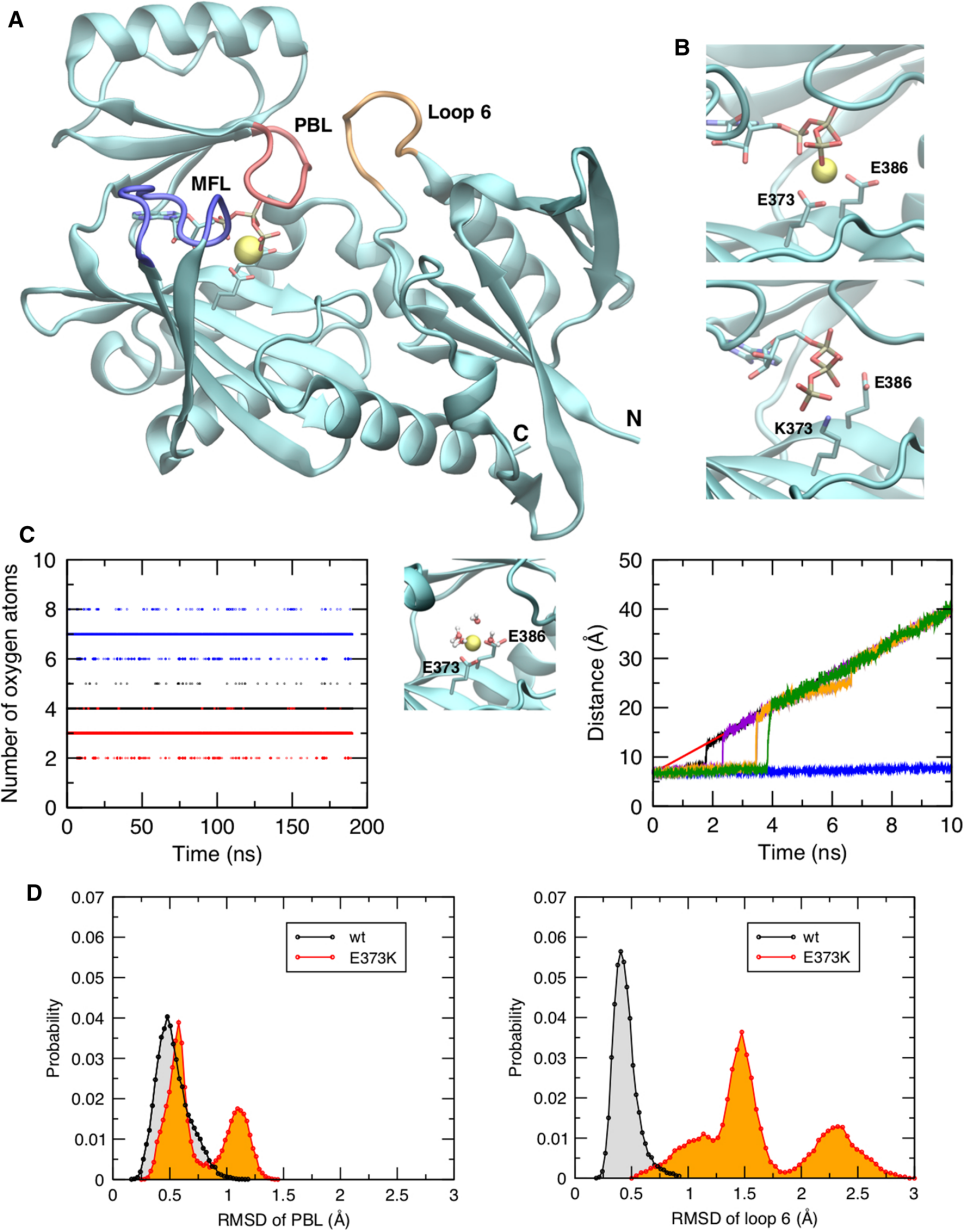
Interdependence of the ATP and Ca^2+^ binding sites in the SynI C-domain. **A** Pictorial representation of the SynI C-domain. The Ca^2+^ atom is represented as a yellow sphere, while the ATP molecule and residues E369 and E373 as sticks. The MFL, PBL, and loop 6 are indicated and colored in blue, red, and orange, respectively. **B** Enlarged view of the ATP binding site in WT (*top*) and E373K (*bottom*) SynI. **C** *Left:* Number of Ca^2+^-coordinating oxygen atoms (within a sphere of 3 Å) during the simulation of WT SynI without ATP, from water (black dots), E373 and E386 (red dots), and total (blue dots). *Middle:* Snapshot extracted from the simulation showing the Ca^2+^ atom (yellow sphere) coordinated by the oxygen atoms from E373, E386, and four water molecules. *Right:* Distance of Ca^2+^ from the main binding site in pulling simulations without ATP (black, purple, orange, brown, and green lines) and with ATP (blue line, representative trajectory); the red line shows the target distance of the ion from the starting site, linearly increasing during the simulation time. **D** Histograms of RMSD values for the PBL (*left*) and the loop 6 (*right*) in WT and E373K SynI from the corresponding simulations

We have previously shown that ATP can bind to SynI also in the absence of Ca^2+^, questioning the classical view that Ca^2+^ binding solely serves to regulate ATP binding [62]. Here, we first addressed the possibility that Ca^2+^ and ATP bind independently of each other to the SynI C-domain and that Ca^2+^ binding plays a further functional role in addition to enhancing ATP binding. Figure 1A shows the structure of the SynI C-domain together with the details of the ATP binding site in SynI^WT^ and in the simulated model of the SynI^E373K^ mutant (Fig. 1B, *top* and *bottom panels*, respectively). In the SynI^WT^ protein, the binding pocket is covered by the MFL that contacts ATP and prevents water access. The MFL, together with the phosphate binding loop (PBL) and loop 6 (Fig. 1A), also contains residues that are relevant for the formation of SynI oligomers. In the SynI^WT^ structure, the Ca^2+^ ion is coordinated by all three ATP phosphate groups, according to the second most observed geometry in experimentally determined structures of proteins containing ATP and a divalent cation [49]. Oxygen atoms from glutamic acid residues E373 and E386 also coordinate Ca^2+^ (Fig. 1B, *top panel*).

In our model of the SynI^E373K^ mutant, Ca^2+^ is absent and the ATP molecule interacts with the substituting lysine (Fig. 1B; *bottom panel*). We next asked whether a Ca^2+^ ion can bind also in the absence of ATP to the main ATP-Ca^2+^ binding site of the SynI^WT^ C-domain revealed in the crystal structure. Thus, we simulated the SynI^WT^ monomer after removing the ATP molecule and leaving the Ca^2+^ ion at the site. We observed that, along the entire simulated trajectory (200 ns), the ion remained stably tethered at the same site, tightly coordinated by E373 and E386 and an average number of four water molecules (Fig. 1C; *left* and *middle panels*). Indeed, when ATP is not present, although the MFL loop remains closed over the binding pocket as in SynI^WT^ [62], more water molecules can access the site with respect to the WT trajectory, thus providing coordinating oxygen atoms to the Ca^2+^ ion.

We then investigated how ATP affects the strength of Ca^2+^ binding to SynI. To this purpose, we ran several simulations in which we pulled the Ca^2+^ ion away from the main site by applying an external force to linearly increase the distance (we applied a harmonic restraint, or a “spring”, on the distance with a linearly increasing target; see “Materials and methods” section). Five simulations were carried out with ATP bound, and five without ATP bound, keeping the force constant. We observed that, while in the SynI-noATP simulations Ca^2+^ moved away from the binding site (Fig. 1C; *right panel*), in the SynI-ATP simulations the Ca^2+^-ATP complex remained tightly tethered at the main binding site (Fig. 1C; blue line in the *right panel*). Thus, being in complex with ATP increases the strength of Ca^2+^ interaction with SynI, indicating that Ca^2+^ and ATP can reciprocally enhance the binding of the respective partner to the binding site [62]. We previously demonstrated that mutations of the ATP binding site in SynI affected its oligomerization dynamics. To examine the possible consequences of E373K mutation on the formation of SynI tetramers, we analyzed the behavior of the tetramer interface residues during the MD simulations of SynI^WT^ and SynI^E373K^ presented in Orlando et al. [62]. We observed that, although ATP remains at the binding site (Fig. 1B, *bottom panel*) and the MFL is closed over the pocket, the mutation enhances conformational disorder in the PBL (Fig. 1D, *left panel*) and in the loop 6 (Fig. 1D, *right panel*). Because these segments contain residues that establish direct contacts between monomers to form the tetramer, their distortion results in the perturbation of the interactions that stabilize the oligomers and are involved in SV clustering.

### Expression of SynI^E373K^ in a SynI KO background increases the frequency of miniature postsynaptic currents only in excitatory synapses

**Fig. 2:**
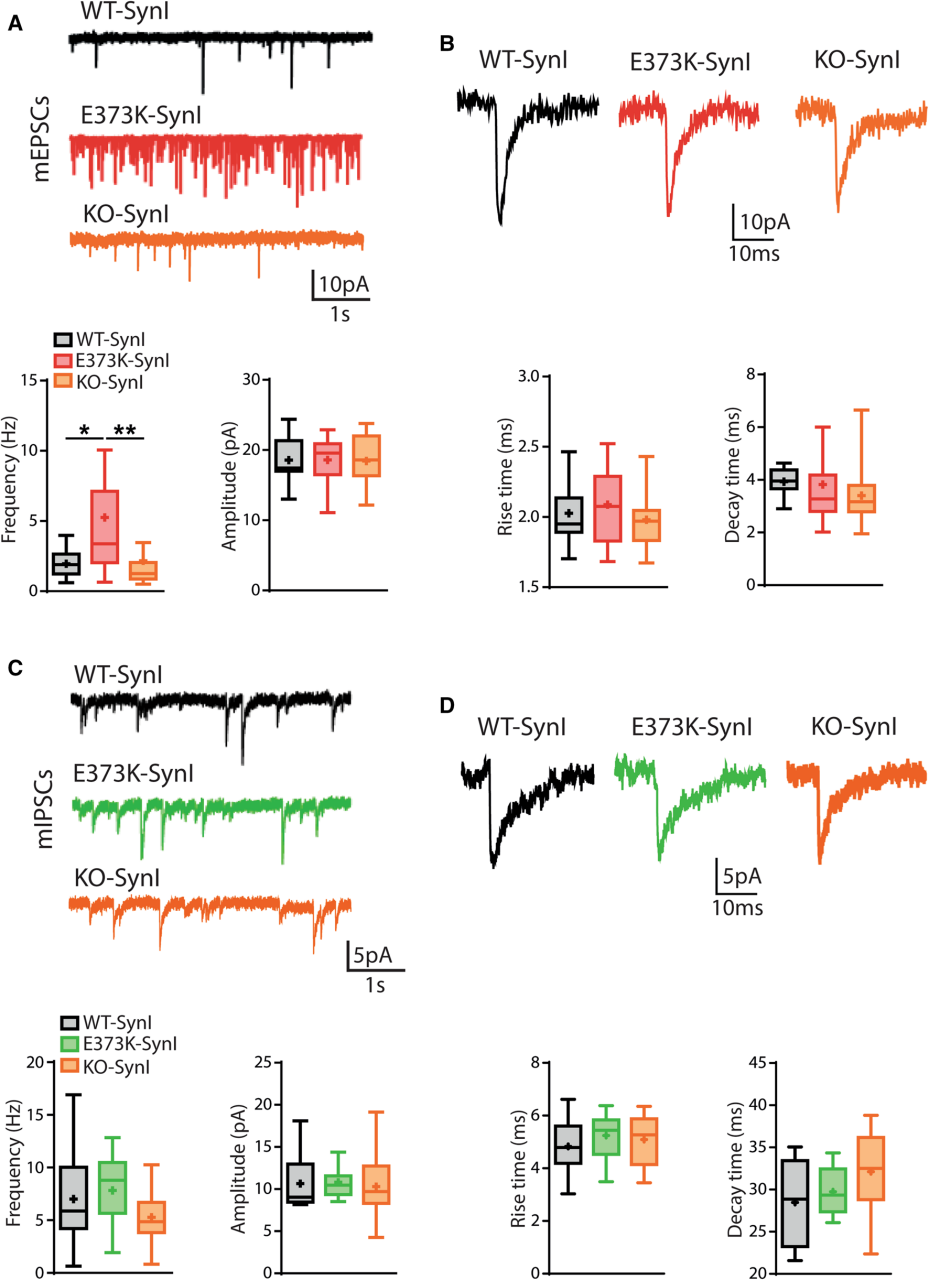
Expression of the SynI^E373K^ mutant selectively increases mEPSC frequency. **A**, **B** mEPSCs. *Top panels:* Representative traces recorded at −70 mV in 14 DIV SynI^KO^ hippocampal neurons expressing empty vector (orange), SynI^WT^ (black), or SynI^E373K^ (red) (**A**) and single events in an expanded time scale (**B**). *Bottom panels:* Box plots of mEPSC frequency and amplitude (**A**) and rise and decay times obtained by fitting individual currents (**B**). **C**, **D** mIPSCs. *Top panels:* Representative traces recorded at −70 mV in 14 DIV SynI^KO^ hippocampal neurons expressing empty vector (orange), SynI^WT^ (black), or SynI^E373K^ (red) (**C**) and single events in an expanded time scale (**D**). *Bottom panels:* Box plots of mEPSC frequency and amplitude (**C**) and rise and decay times obtained by fitting individual currents (**D**). Excitatory synapses: *n* = 19, 20, 20; Inhibitory synapses: *n* = 14, 17, 16; for SynI^KO^, SynI^WT^, and SynI^E373K^, respectively from *n* = 3 independent neuronal preparations. *p < 0.05; **p < 0.01; Kruskal–Wallis/Dunn’s tests

Based on the suggestions by MD simulations that Ca^2+^ binds with high affinity at the ATP binding site and affects the tetramerization surface, we proceeded to physiologically test the SynI^E373K^ mutant that abolishes the main Ca^2+^ binding, without affecting the binding of ATP [62]. To address the functional role of Ca^2+^ binding to SynI using lentiviral vectors and low-density primary hippocampal neurons from SynI KO mice, we expressed: (i) a reporter-containing empty vector for the control KO group (SynI^KO^; (ii wild type mCherry-SynI (SynI^WT^); and (iii) mCherry-SynI mutant in the Ca^2+^ binding site (SynI^E373K^). Analysis of the fluorescent reporters showed that the vast majority of the neurons (SynI^KO^: 96.41 ± 0.34; SynI^WT^: 95.13 ± 0.25; SynI^E373K^: 94.74 ± 0.27) were transduced with comparable expression levels. We first investigated whether the expression of SynI^E373K^ could alter the physiological properties of excitatory (Fig. 2A, B) and inhibitory (Fig. 2C, D) miniature postsynaptic currents (mEPSCs and mIPSCs, respectively) as compared to SynI^WT^ and SynI^KO^. The expression of the SynI^E373K^ mutant markedly increased the frequency of mEPSCs with respect to Syn^KO^ neurons or neurons rescued with Syn^WT^, while the amplitude was not affected (Fig. 2A*; lower panels*).

On the contrary, no changes in the frequency and amplitude of mIPSCs were observed across the three genotypes (Fig. 2C; *lower panels*). The analysis of the rise and decay times revealed no significant changes in the kinetics of both mEPSCs (Fig. 2B) and mIPSCs (Fig. 2D) across the three genotypes. The results uncover a strong and selective effect of the SynI Ca^2+^ mutant on the mEPSC frequency with preservation of the quantal size, thus excluding the presence of secondary postsynaptic effects. The data also confirm that the *Syn1*deletion per se does not significantly affect the properties of mPSCs, as previously reported [14].

### SynI^E373K^ does not influence the density of excitatory and inhibitory synapses

**Fig. 3:**
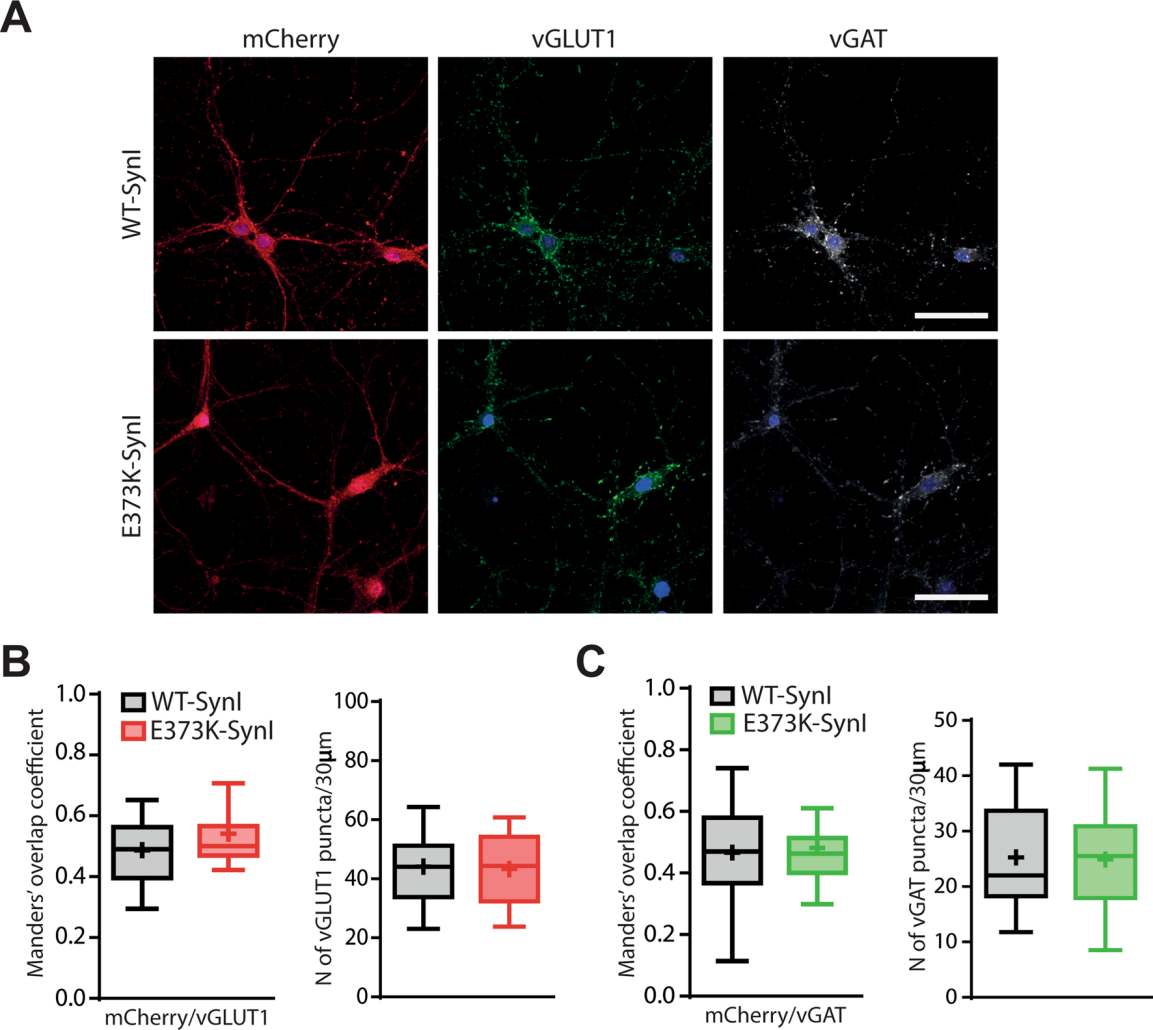
The SynI^E373K^ mutant does not affect excitatory and inhibitory synaptic density. **A** Representative images of SynI^WT^ and SynI^E373K^ transduced hippocampal neurons at 14 DIV triple stained for mCherry (red), vGLUT1 (gray), and vGAT (green). Nuclei were stained with Hoechst. Scale bar, 50 μm. **B** Excitatory synapses. Manders’ colocalization coefficient of vGLUT1- and mCherry-positive synapses (*left*) and linear density of vGLUT1-positive boutons on proximal dendrites of postsynaptic cells (*right*). **C** Inhibitory synapses. Manders’ colocalization coefficient of vGAT- and mCherry-positive synapses (*left*) and linear density of vGAT-positive boutons on proximal dendrites of postsynaptic cells (*right*). Data are shown as box plots. Synaptic density: *n* = 23 and 20 fields for SynI^WT^ and SynI^E373K^, respectively; Manders’ colocalization coefficient: *n* = 18 and 30 fields for SynI^WT^ and SynI^E373K^, respectively, from *n* = 3 independent cell culture preparations. *p* > 0.05; Mann–Whitney *U*-test/unpaired Student’s *t*-test

An increased frequency of miniature synaptic events is normally attributable to either an increased number of synapses or an increased probability of spontaneous SV fusion. To test the first possibility, we investigated whether the expression of SynI variants could differentially alter the density of excitatory and inhibitory synapses labeled by the presynaptic markers vGLUT1 and vGAT, respectively (Fig. 3A).

The analysis of the Manders’ colocalization coefficient revealed that about 50% of virally expressed Syn isoforms (mCherry-positive puncta) localized at vGLUT1- and vGAT-positive puncta identifying putative inhibitory and excitatory synaptic contacts (Fig. 3B, C; *left panels*). Next, we evaluated the number of excitatory and inhibitory synapses along proximal dendrites by vGLUT1/vGAT double immunostaining of transduced hippocampal neurons and found no significant differences in the density of both excitatory and inhibitory synaptic contacts (Fig. 3B, C; *right panels*). This demonstrates that the development of synaptic connectivity is not significantly affected by the E373K mutation and that the increased mEPSC frequency observed in SynI^E373K^ transduced neurons can be tentatively attributed to an increased probability of spontaneous release.

### SynI^E373K^ increases the nerve terminal resting Ca^2+^ concentration

**Fig. 4:**
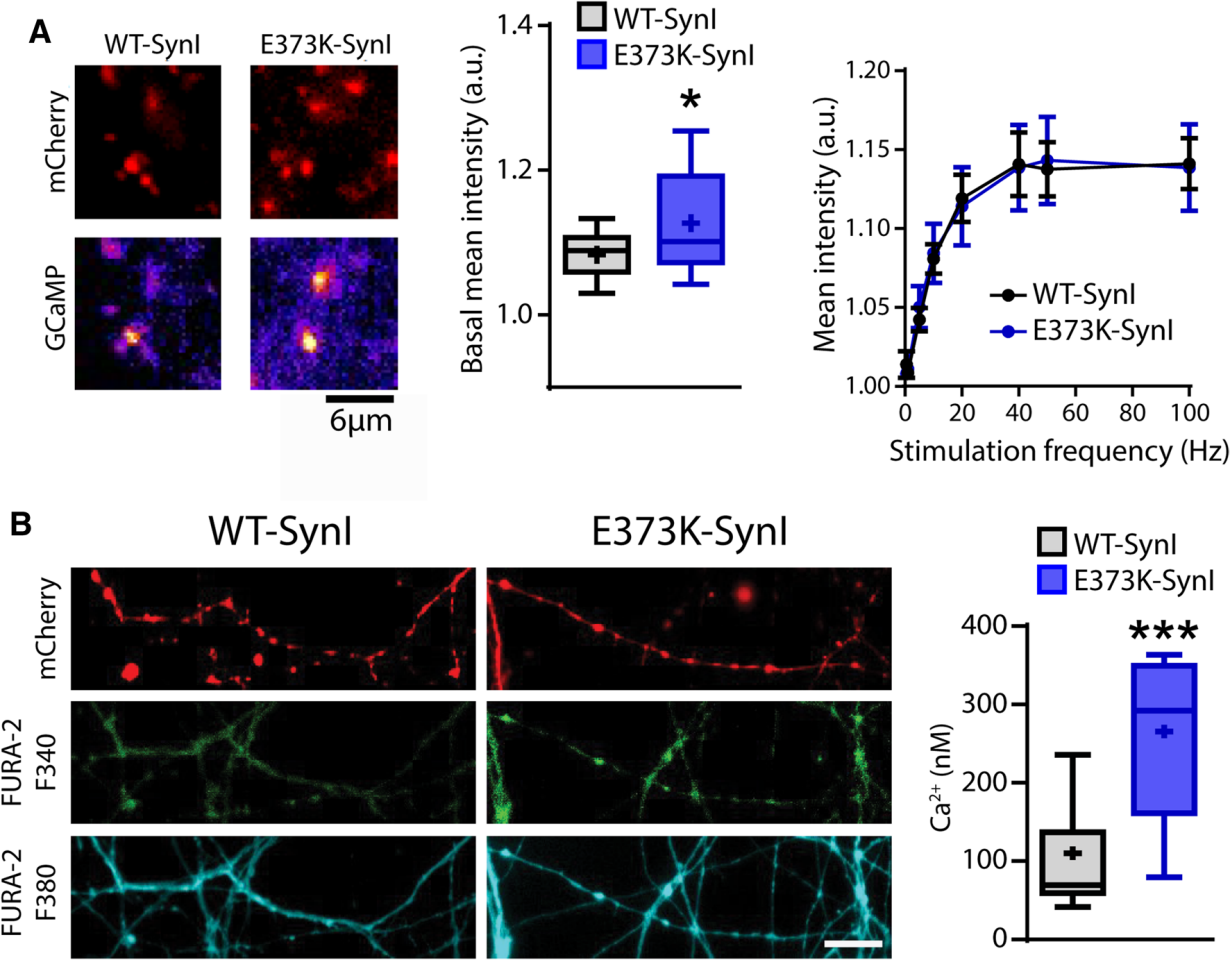
Expression of the SynI^E373K^ mutant is associated with an increase in resting, but not activity-stimulated, [Ca^2+^]_i_. **A** Nerve terminal Ca^2+^ measurements with SynGCaMP6. Primary SynI KO hippocampal neurons were co-transduced with SynGCaMP6 and either SynI^WT^ or SynI^E373K^ tagged with mCherry. *Left:* Representative images of mCherry-positive boutons where the fluorescence intensity was measured. Note the higher SynGCaMP6 fluorescence in boutons expressing SynI^E373K^. Scale bar, 6 μm. *Middle:* Mean (± SEM) integrated resting SynGCaMP6 fluorescence measured at mCherry-positive boutons in SynI KO neurons expressing either SynI^WT^ (black, *n* = 14) or SynI^E373K^ (blue, *n* = 14). *Right:* Mean (± SEM) integrated SynGCaMP6 fluorescence measured at mCherry-positive puncta in response to 2-s electrical field stimulation with 2–200 APs delivered at increasing frequencies (from 0.5 to 100 Hz). *n* = 15 and 14 fields for both SynI^WT^ and SynI^E373K^ from *n* = 3 independent neuronal preparations. *p < 0.05, unpaired Student’s *t*-test. **B** Nerve terminal Ca^2+^ measurements with Fura-2. *Left:* Representative images showing Fura-2 AM-filled neuronal branches from 14 DIV SynI KO hippocampal neurons transduced with either Syn^WT^ or SynI^E373K^ fused to mCherry (red). Scale bar, 10 μm. *Right:* Box plots of basal Ca^2+^ concentrations, measured at mCherry-positive boutons. ***p < 0.001; Mann–Whitney *U*-test; *n* = 16 and *n* = 11 for SynI^WT^ and SynI^E373K^, respectively, from *n* = 3 independent neuronal preparation

It is known that the spontaneous release of SVs that occurs stochastically in nerve terminals is dependent on the resting intraterminal [Ca^2+^]_i_ [44, 45]. Thus, we addressed the possibility that SynI can actively bind and buffer Ca^2+^ in the nerve terminal under resting conditions, thus contributing to the resting [Ca^2+^]_i_. To ascertain whether deletion of the Ca^2+^-binding site on SynI alters resting [Ca^2+^]_i_, we co-transduced SynI KO hippocampal neurons with the SyGCaMP6f and either SynI^WT^ or SynI^E373K^ tagged with mCherry. SyGCaMP6f is an ultra-sensitive (*K*_d_ = 375 nM) genetically encoded fluorescent Ca^2+^ indicator in which GCaMP6f is fused to the cytoplasmic domain of the integral SV protein synaptophysin, allowing specific measurements of presynaptic Ca^2+^ concentrations in the nerve terminal cytosol surrounding SVs [63]. To effectively investigate the effects of SynI^E373K^ expression on intraterminal resting Ca^2+^ concentrations, SyGCaMP6f fluorescence was quantitatively analyzed at mCherry-positive boutons (Fig. 4A, *left*).

Interestingly, under resting conditions, boutons expressing SynI^E373K^ displayed a significantly increased [Ca^2+^]_i_ with respect to presynaptic sites expressing SynI^WT^ (Fig. 4A, *middle*), indicating that SynI^WT^ might function as a high-affinity Ca^2+^-buffer within nerve terminals. This possibility was also supported by the observation that no differences in the amplitude of activity-dependent Ca^2+^ transients were observed upon field stimulation in a wide range of stimulation frequencies (Fig. 4A, *right*). To independently assess the absolute [Ca^2+^]_i_ at the presynaptic compartment, we loaded both SynI^WT^ and SynI^E373K^ transduced SynI KO neurons with the ratiometric Ca^2+^ sensor Fura-2 AM and analyzed Fura-2 fluorescence at the level of mCherry-positive puncta. Under resting conditions, SynI^E373K^-positive boutons showed a significant increase in the intraterminal [Ca^2+^]_i_ compared to SynI^WT^-positive boutons (110.1 ± 18.78 nM vs 265.4 ± 30.02 nM; Fig. 4B), confirming the semiquantitative data obtained with SyGCaMP6f.

### The effects of SynI^E373K^ on miniature excitatory postsynaptic currents are rescued by intracellular Ca^2+^ chelators

The higher resting [Ca^2+^]_i_ in SynI^E373K^-expressing neurons suggests that the increased frequency of mEPSCs, in the presence of unchanged synaptic density, is attributable to an increased probability of spontaneous release.

**Fig. 5:**
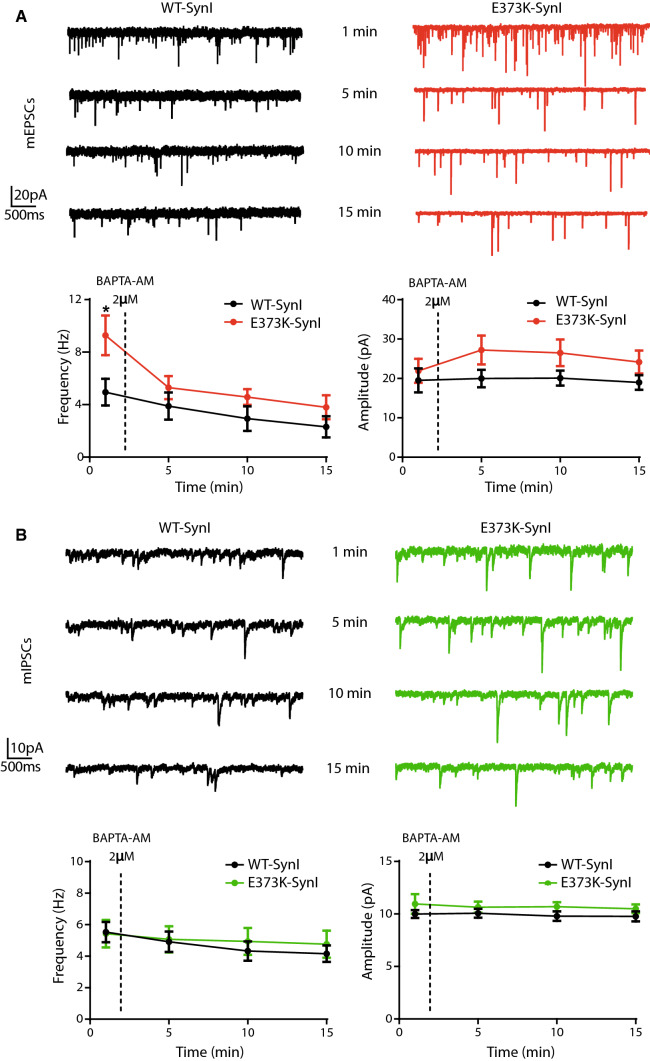
The enhancement of mEPSC frequency by the SynI^E373K^ mutant is suppressed by BAPTA. **A** mEPSCs. *Upper panels:* Representative traces recorded at −70 mV in 14 DIV SynI KO hippocampal neurons transduced with either SynI^WT^ (black) or SynI^E373K^ (red) before and 5, 10, and 15 min after the addition of BAPTA-AM (2 μM). *Lower panels:* Time course of the mean (± SEM) changes in frequency (left) and amplitude (right) of mEPSCs. *n* = 5 and 8 for SynI^WT^ and SynI^E373K^, respectively. **B** mIPSCs. *Upper panels:* Representative traces recorded at −70 mV in 14 DIV SynI KO hippocampal neurons transduced with either SynI^WT^ (black) or SynI^E373K^ (green) before and 5, 10, and 15 min after the addition of BAPTA-AM (2 μM). *Lower panels:* Time course of the mean (± SEM) changes in frequency (*left*) and amplitude (*right*) of mIPSCs. *n* = 12 and 14 for SynI^WT^ and SynI^E373K^, respectively. Data are obtained from *n* = 3 independent neuronal preparations. *p < 0.05; two-way ANOVA/Bonferroni’s tests

To obtain independent evidence on the mechanism and its specificity for excitatory synapses, we recorded mPSC events before and after the treatment with BAPTA-AM, a cell-permeable, fast Ca^2+^-chelator. Both excitatory and inhibitory mPSCs were initially recorded in the control extracellular solution for 2 min. After the addition of BAPTA-AM (2 µM) to the external solution, mPSCs were recorded for a further 15 min and analyzed in three consecutive time windows of 5 min. Strikingly, the increased mEPSC frequency that characterized SynI KO neurons transduced with SynI^E373K^ was totally occluded by BAPTA-AM treatment, while mEPSC amplitude resulted similarly unchanged by BAPTA-AM in both genotypes (Fig. 5A). As expected, the BAPTA-AM treatment did not differentially affect SynI^WT^ and SynI^E373K^ expressing inhibitory synapses (Fig. 5B). Taken together, the data indicate that an alteration in the resting presynaptic Ca^2+^ homeostasis due to lack of Ca^2+^-binding to SynI could be the mechanistic cause of the specific enhancement of mEPSC frequency.

### SynI^E373K^ reduces the amplitude of excitatory, but not inhibitory, evoked postsynaptic currents

**Fig. 6:**
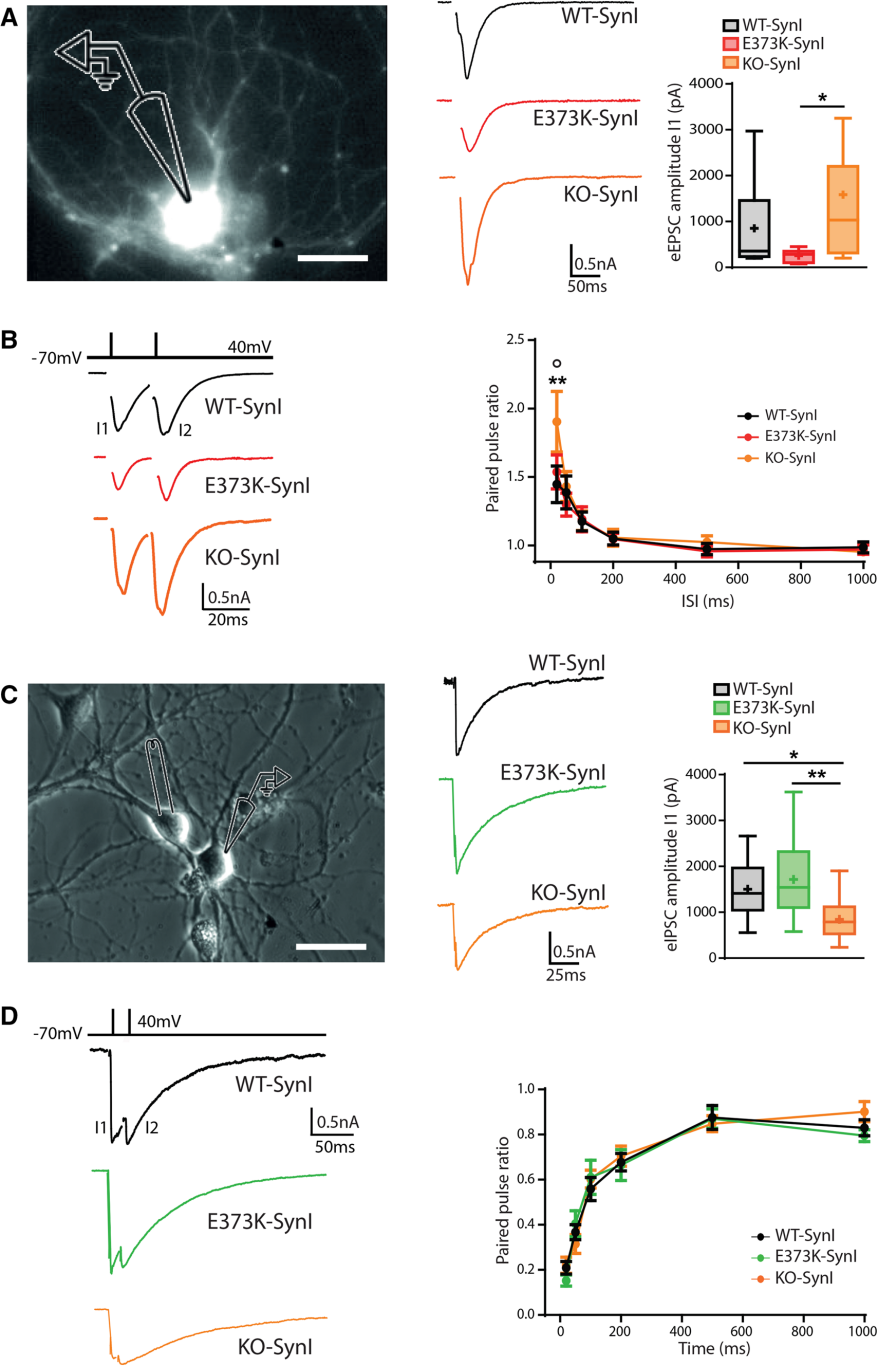
Mutant SynI^E373K^ decreases evoked EPSC, but not eIPSC, amplitude. **A, B** eEPSCs. **A** *Left:* Representative image of patched autaptic neuron for the study of eEPSCs at excitatory synapses. Scale bar, 15 μm. *Right:* Representative traces of single-pulse protocols applied to excitatory SynI KO synapses transduced with control vector (SynI^KO^, orange), SynI^WT^ (black) or SynI^E373K^ (red) and box plots of the eEPSC amplitude (*n* = 10, 14, 13 for SynI^KO^, SynI^WT^, and SynI^E373K^, respectively). **B** *Left:* Representative traces of paired-pulse protocols applied to excitatory synapses of the three genotypes. *Right:* Paired-pulse ratio (means ± SEM) of excitatory synapses transduced with control vector (SynI^KO^, orange), SynI^WT^ (black), or SynI^E373K^ (red) at interpulse intervals ranging from 20 ms to 1 s (*n* = 11–14, 20–21, 13–18 for SynI^KO^, SynI^WT^ and SynI^E373K^, respectively). **C**, **D** eIPSCs. **C** *Left:* Representative images of low-density neuron culture for the study of eIPSCs at inhibitory synapses. Scale bar, 50 μm. *Right:* Representative traces of single-pulse protocols applied to inhibitory SynI KO synapses transduced with control vector (SynI^KO^, orange), SynI^WT^ (black), or SynI^E373K^ (green) and box plots of the eIPSC amplitude (*n* = 16, 13, 15 for SynI^KO^, SynI^WT^ and SynI^E373K^, respectively). **D** *Left:* Representative traces of paired-pulse protocols applied to inhibitory synapses of the three genotypes. *Right:* Paired-pulse ratio (means ± SEM) of inhibitory synapses transduced with control vector (SynI^KO^, orange), SynI^WT^ (black) or SynI^E373K^ (red) at interpulse intervals ranging from 20 ms to 1 s (*n* = 16, 13, 15 for SynI^KO^, SynI^WT^ and SynI^E373K^, respectively). Data are obtained from *n* = 3 independent neuronal preparations. *p < 0.05, **p < 0.01; Kruskal–Wallis/Dunn’s tests (**A**) or one-way ANOVA/Tukey’s tests (**C**). °*p* < 0.05 SynI^E373K^ *vs* SynI^KO^; **p < 0.01 SynI^WT^ *vs* SynI^KO^; two-way ANOVA/Tukey’s test (**B**, **D**)

Next, we investigated whether the expression of SynI^E373K^ could differentially alter evoked neurotransmitter release by excitatory and inhibitory synapses. Since SynI plays pre- and post- docking roles in synaptic transmission [11, 50], we analyzed single-pulse evoked excitatory (eEPSCs) and inhibitory (eIPSCs) synaptic currents in SynI^WT^- and SynI^E373K^-expressing SynI KO neurons. eEPSCs were studied in primary autaptic neurons by depolarizing the cell membrane of the excitatory neuron to + 40 mV for 0.5 ms (Fig. 6A; *left panel*). Excitatory synapses expressing the SynI^E373K^ mutant showed a significant reduction in the amplitude of eEPSCs in response to the first pulse with respect to SynI^WT^ expressing synapses (Fig. 6A; *right panel*). The different amplitude of eEPSCs brought us to investigate the response to paired-pulse stimulation, in which both excitatory synapses were subjected to two consecutive stimuli at interstimulus intervals (ISI) ranging between 20 ms and 1 s (Fig. 6B; *left panel*). Excitatory synapses displayed facilitation at short ISIs that vanished at longer stimulation intervals. As previously reported [14, 70], SynI^KO^ excitatory synapses displayed increased facilitation at short ISIs that was normalized by the expression of SynI^WT^. However, no differences in the expression of this short-term plasticity paradigm were found between SynI^WT^ and SynI^E373K^ (Fig. 6B, *right panel*).

eIPSCs were studied in low-density neuronal cultures, in which the presynaptic neuron was extracellularly stimulated by micromanipulating the stimulating electrode near the putative presynaptic neuron (Fig. 6C; *left panel*). No changes were observed in the amplitude of eIPSCs evoked in SynI^E373K^-transduced inhibitory synapses as compared with SynI^WT^ synapses (Fig. 6C; *right panel*). Inhibitory synapses exhibited a characteristic depression in response to paired-pulse stimulation that was particularly intense at shorter ISIs and attenuated at longer stimulation intervals (Fig. 6D; *left panel*). This rescue from paired-pulse depression was closely similar in inhibitory synapses from the three genotypes (Fig. 6D; *right panel*).

The decreased amplitude of eEPSC responses indicates that the impaired Ca^2+^-buffering by mutated SynI under resting conditions does not play a role in the stimulus-dependent Ca^2+^ entry in the nanodomains of the active zones, suggesting the involvement of an alternative mechanism.

### The reduction of eEPSC amplitude by SynI^E373K^ is attributable to a decrease in the size of the RRPsyn

To identify the quantal parameters of release affected by SynI_E373K_ mutation, we performed cumulative ePSC amplitude analysis in excitatory and inhibitory SynI KO neurons transduced with empty vector (SynI^KO^), SynI^WT^ or SynI^E373K^. This method analyzes the cumulative amplitude profile during high- frequency trains of stimuli [5, 73], allowing to extract the values of P_r_ and RRP_syn_.

**Fig. 7:**
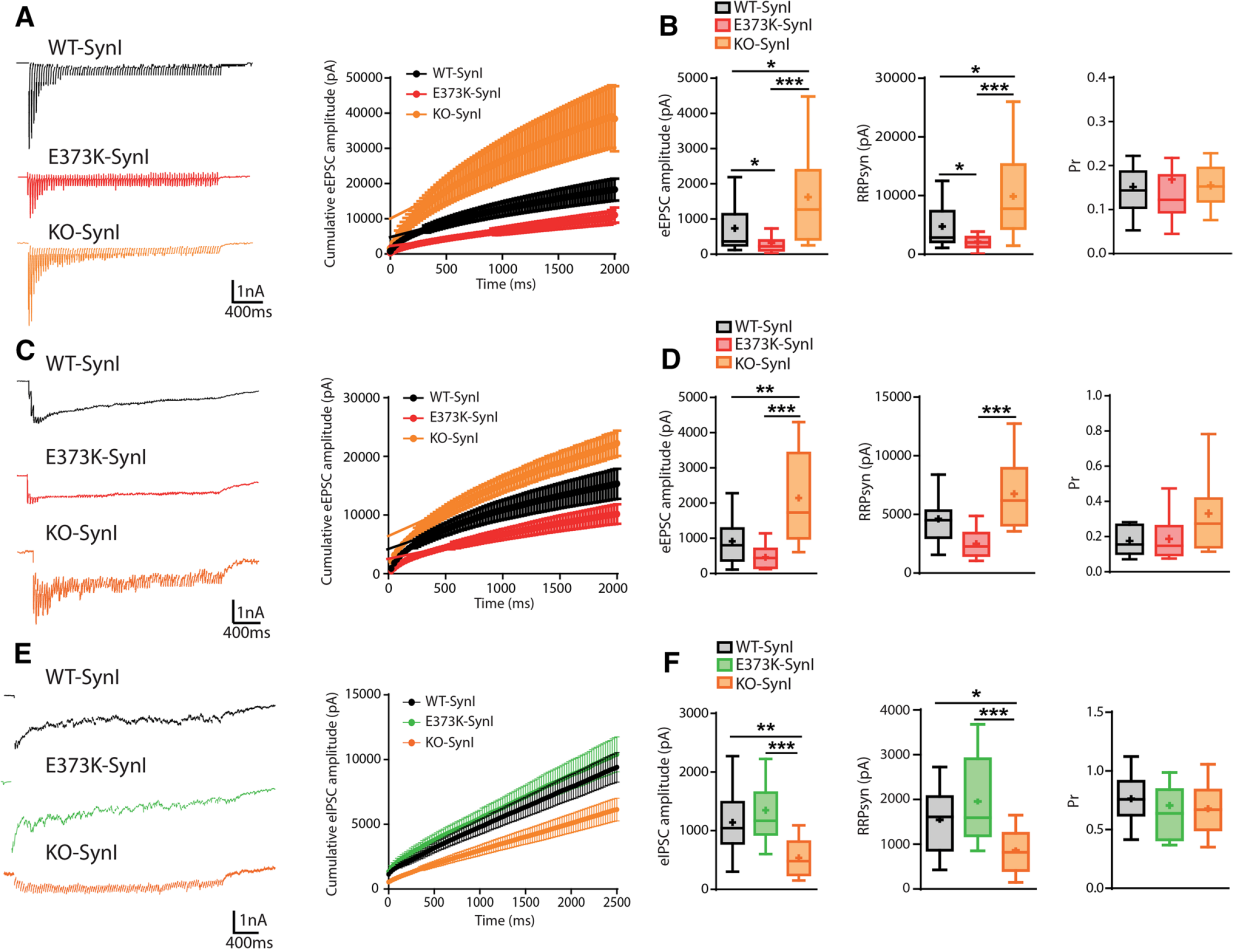
The SynI^E373K^ mutant decreases the RRP size for synchronous release in excitatory, but not inhibitory, synapses. **A**–**D** Excitatory synapses. **A**, **C** *Left:* Representative traces showing the 2-s stimulation protocol @ 40 Hz used to estimate the quantal properties of synchronous release in the absence (**A**) or presence (**C**) of CTZ (60 µM) in excitatory SynI KO autapses expressing the control vector (SynI^KO^; orange), SynI^WT^ (black) or SynI^E373K^ (red). *Right:* The corresponding cumulative curves of the eEPSC amplitude during the stimulation train in the absence or presence of CTZ are shown as means ± SEM together with the linear regression fit to their linear phases (*right*). **B**, **D** Cumulative amplitude analysis of quantal parameters in excitatory autapses in the absence (**B**) or presence (**D**) of CTZ. Box plots of the amplitude of the first EPSC in the train (*left*), RRP size for synchronous release (RRP_syn_; *middle*) and release probability (P_r_; *right*). Without CTZ: *n* = 17, 29, 28; with CTZ: *n* = 12, 12, 9; for SynI^KO^ SynI^WT^, and SynI^E373K^, respectively. **E**, **F** Inhibitory synapses. **E** Representative traces (*left*) showing the 2.5-s stimulation protocol @ 40 Hz used to estimate the quantal properties of synchronous release in inhibitory SynI KO synapses expressing the control vector (SynI^KO^; orange), SynI^WT^ (black) or SynI^E373K^ (green) and corresponding cumulative curves of the eIPSC amplitude during the stimulation train (*right*). **F** Box plots of the amplitude of the first IPSC in the train (*left*), RRP size for synchronous release (RRP_syn_; *middle*) and release probability (P_r_; *right*). *n* = 15, 16, 14 for SynI^KO^ SynI^WT^, and SynI^E373K^, respectively. For further details, see “Materials and methods” section. Data are obtained from *n* = 3 independent neuronal preparations. *p < 0.05; **p < 0.01; ***p < 0.001; Kruskal–Wallis/Dunn’s tests (**B**) or one-way ANOVA/Tukey’s tests (**D**, **F**)

When excitatory neurons were challenged with a 40 Hz train for 2 s, a significant depression of eEPSCs became apparent, irrespective of the amplitude of the first current in the train (Fig. 7A; *left panel);* the method assumes that *P*_r_ during the train approaches unity and that depression during the steady-state phase is limited by a constant recycling of SVs. Accordingly, the cumulative profile showed a rapid rise followed by a slower linear increase, reflecting the equilibrium between depletion and constant replenishment of the RRP (Fig. 7A; *right panel*). Consistent with previous reports [14], the eEPSC amplitude of SynI^KO^ excitatory synapses was larger than that of SynI^WT^ synapses, due to a pure increase in the RRP_syn_ size. The decreased eEPSC amplitude of the SynI^E373K^ mutant (see also Fig. 6A) was entirely attributable to a significant drop, approximately of the same extent, in size of the RRP_syn_, in the absence of changes in the P_r_ of evoked release (Fig. 7B) and consistent with the lack of effect on PPR at short time intervals (see Fig. 6B). To verify if the drop in both amplitude and RRP_syn_ of eEPSCs due to the expression of the SynI^E373K^ mutant was strictly dependent on a presynaptic impairment or the result of a postsynaptic receptor deficit, we performed the same experiments in the presence of CTZ to selectively block AMPA receptor desensitization due to the intense glutamate release [15], Fig. 7C). Interestingly, the desensitization blockade did not change the quantal parameters reported above (Fig. 7D), confirming that the SynI^E373K^ mutant negatively affects excitatory transmission by acting at the presynaptic level. The same cumulative amplitude analysis was performed on inhibitory synapses, using a 40 Hz train for 2.5 s (Fig. 7E). While the decreased eIPSC amplitude of SynI^KO^ synapses was associated with a corresponding decrease in the RRP_syn_, as previously reported [14], the lack of effect of SynI^E373K^ on the eIPSC amplitude was confirmed by the absence of significant changes in the quantal parameters of synchronous inhibitory transmission (Fig. 7F). Taken together, the data suggest that the SynI^E373K^ mutant affects the activity-dependent refilling of the RRP from the RecP specifically in excitatory synapses.

### SynI^E373K^ impairs the recovery from synaptic depression in both excitatory and inhibitory synapses

**Fig. 8:**
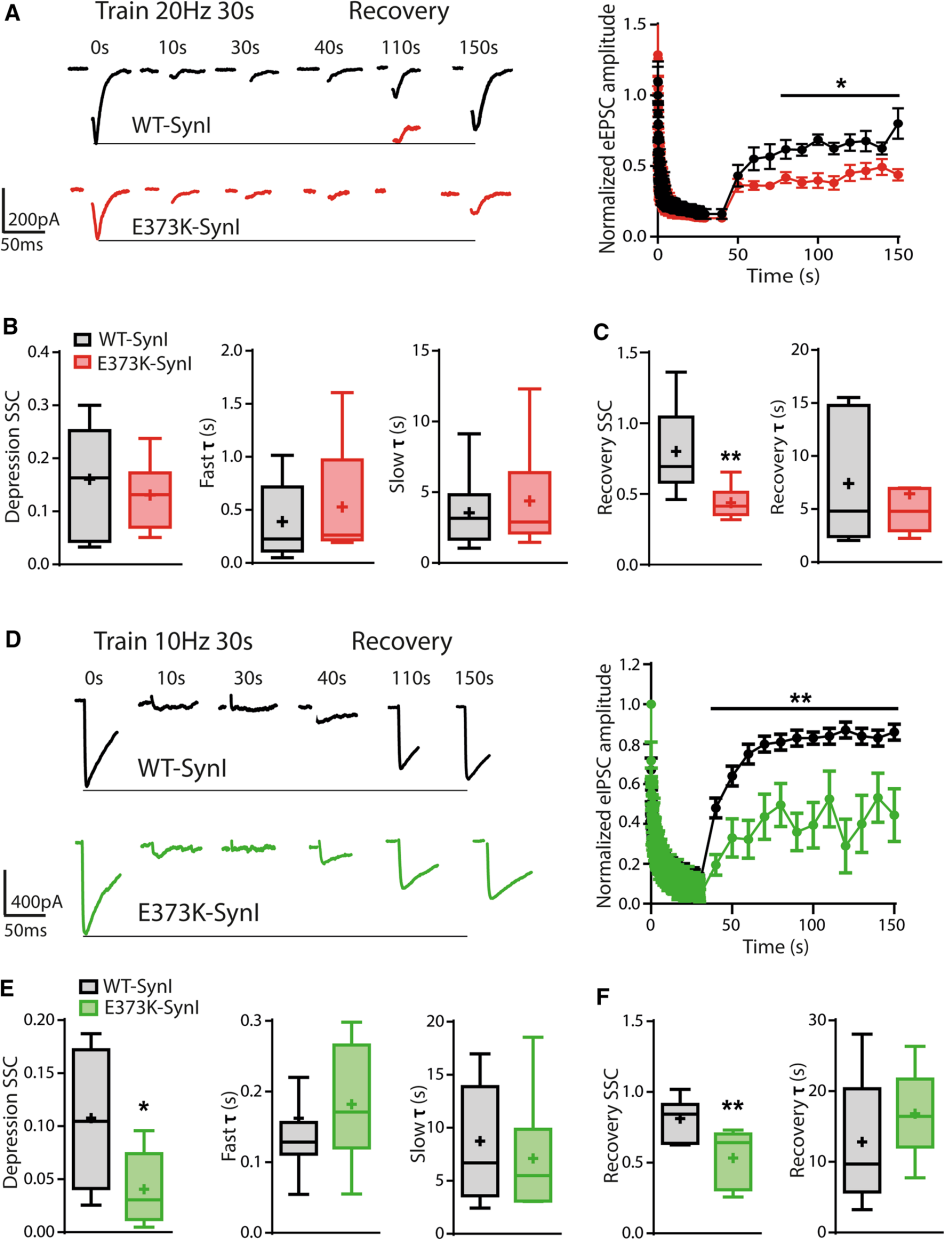
The SynI^E^^373K^ mutant impairs recovery from depression in both excitatory and inhibitory synapses. **A**–**C** Excitatory synapses. Excitatory SynI KO autaptic neurons were transduced with either SynI^WT^ (black) or SynI^E373K^ (red). **A** *Left:* Representative traces of eEPSCs evoked by stimulating neurons with high**-**frequency trains lasting 30 s at 20 Hz. *Right:* The progressive decay of the eEPSC amplitude (means ± SEM) during the stimulation train and the subsequent recovery from depression upon return of the stimulation frequency to 0.1 Hz are plotted as a function of time from the beginning of the train (*n* = 9 and 8 for SynI^WT^ and SynI^E373K^, respectively). **B** Box plots of the depression steady-state current (SSC, *left*) and of the fast (*middle*) and slow (*right*) time constants of depression (τ). **C** Box plots of the SSC (*left*) and τ (*right*) of recovery after the stimulation train. **D**–**F** Inhibitory synapses. Inhibitory SynI KO neurons were transduced with either SynI^WT^ (black) or SynI^E373K^ (green). **D** *Left:* Representative traces of eIPSCs evoked by stimulating neurons for 30 s at 20 Hz. *Right:* The progressive decay of the eIPSC amplitude (means ± SEM) during the stimulation train and the recovery from depression are plotted as a function of time (*n* = 9 and 8 for SynI^WT^ and SynI^E373K^, respectively). (**E**) Box plots of the depression steady-state current (SSC, *left*) and of the fast (*middle*) and slow (*right*) time constants of depression (τ). (**F**) Box plots of the SSC (*left*) and τ (*right*) of recovery after the stimulation train. Data are from *n* = 3 independent neuronal preparations. *p < 0.05; **p < 0.01; Mann–Whitney *U*-test (**A**) or unpaired Student’s *t*-test (**C**–**F**)

Central synapses in mice lacking one or more Syn isoforms display a marked decrease in SV density, reflecting a depletion of the RecP and changes in short-term, but not long-term, synaptic plasticity [34, 48, 69, 70, 76, 78, 80]. Accordingly, most of the available data indicate that Syn deletion enhances synaptic depression during repetitive stimulation [25, 34]. Excitatory and inhibitory synapses of SynI KO neurons transduced with either SynI^WT^ or SynI^E373K^ were subjected to a prolonged high-frequency stimulation (HFS). The progressive decay of the ePSCs amplitude during the train and the subsequent recovery from depression upon returning the stimulation frequency to 0.1 Hz were exponentially fitted and analyzed for the kinetic parameters and the steady-state current of depression and recovery. During sustained HFS (30 s @ 20 Hz), SynI^E373K^ excitatory synapses presented no significant changes in the depression steady-state current (SSC) and in the slow and fast time constants of depression (Fig. 8A, B). However, they showed a significant impairment in the recovery after depression with an almost two-fold difference in the recovery SSC with respect to SynI^WT^, in the absence of changes in the time constant of recovery (Fig. 8A, C). Under sustained HFS (30 s @ 10 Hz), SynI^E373K^ inhibitory synapses showed a significantly lower depression SSC than SynI^WT^ inhibitory synapses, with no significant differences in the slow and fast time constants of depression (Fig. 8D, E). In addition, inhibitory synapses displayed an impaired recovery after depression similar to that observed at excitatory synapses: the first response after the stimulus train was markedly smaller than in SynI^WT^ synapses and the recovery SSC was twofold lower, with no change in the time constant of recovery (Fig. 8D, F).

These results suggest that the SynI^E373K^ mutant strongly impairs the recovery from depression in both excitatory and inhibitory synapses and the steady state of depression in inhibitory synapses. This suggests that Ca^2+^ binding to SynI plays a role in the dynamics of SV pools and in SV mobilization during and after sustained HFS in both types of synapses.

### SynI^E373K^ impairs SV recycling and slows-down the kinetics of RRP refilling

**Fig. 9:**
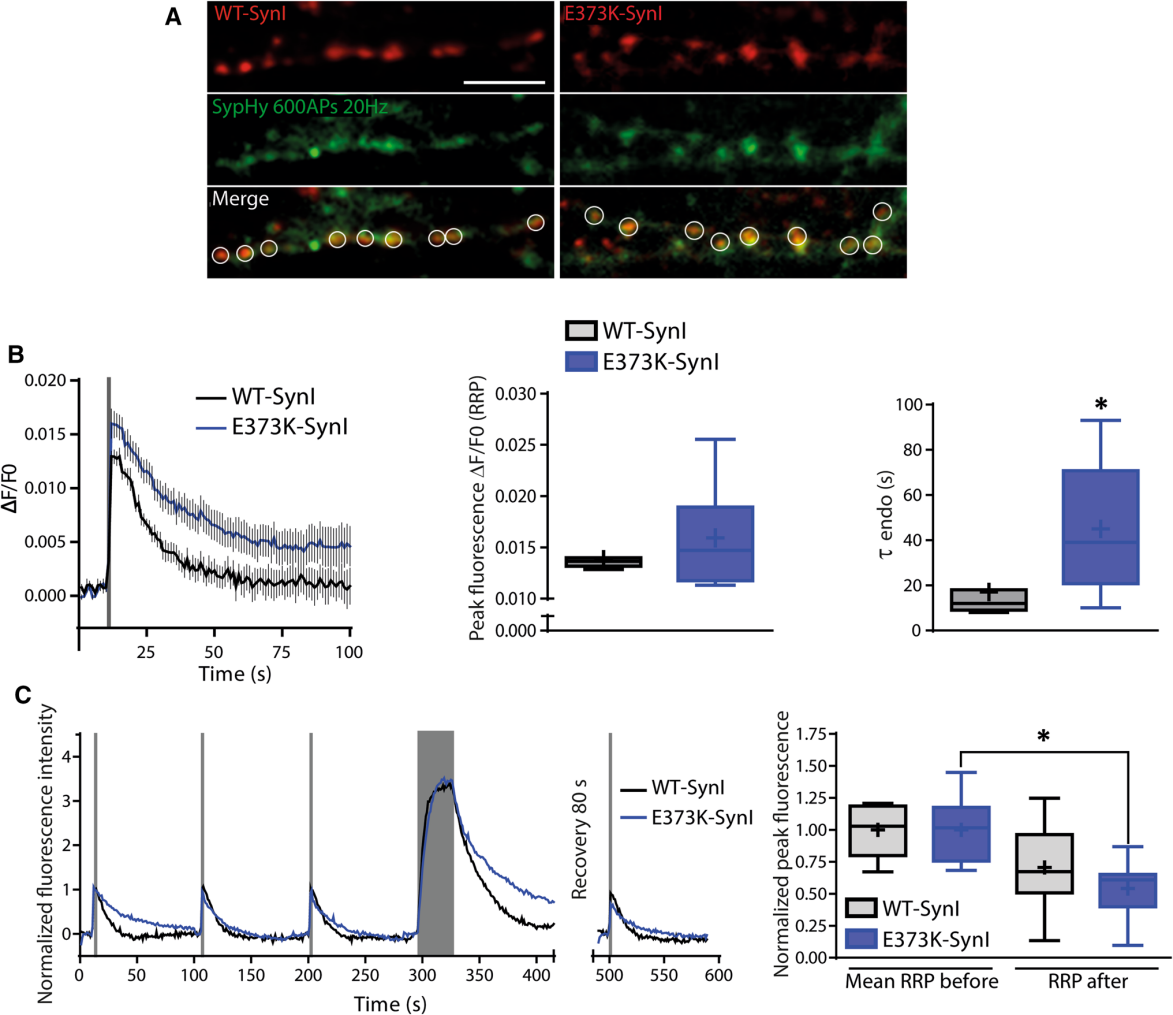
The recovery from the synaptic activity is altered in nerve terminals expressing the SynI^E373K^ mutant. **A** Representative images showing axonal branches from 21 DIV SynI KO hippocampal neurons transduced with sypHy (green) and either Syn^WT^ (*left*) or SynI^E373K^ (*right*) fused to mCherry (red) during a 600 AP stimulation @ 20 Hz. White regions of interest (ROI) label co-transduced synapses. Scale bar, 3 µm. **B** *Left:* Ensemble average traces of sypHy fluorescence plotted for Syn^WT^ (black) and SynI^E373K^ (blue). Stimulation (20 APs@100 Hz) is indicated by the gray bar. Data are means ± SEM of *n* = 7 and 11 coverslips for Syn^WT^ and SynI^E373K^, respectively. *Middle:* Box plot of peak fluorescence at the end of the stimulus in the two genotypes. *Right:* Box plot of time constant of endocytosis (τ_endo_), evaluated by fitting the fluorescence decay after stimulation with a mono-exponential function. *p < 0.05, Mann–Whitney’s *U*-test. **C** *Left:* Representative traces from Syn^WT^ (black) and SynI^E373K^ (blue) expressing synapses stimulated with three RRP trains (20 APs@100 Hz, thin gray bars), subjected to a long-lasting stimulation (600 APs@20 Hz, thick gray bar) and challenged with a further RRP stimulation. Traces were normalized to the first stimulus. *Right:* Box plot showing the average peak fluorescence for the three consecutive RRP stimulations before the long-lasting train, and the response to an additional RRP stimulation performed after the long-lasting train. Data are from *n* = 9 coverslips per genotype. *p < 0.05, one-way ANOVA/Tukey’s tests

To further investigate the mechanisms of defective recovery after the stimulus in SynI^E373K^ synapses, we performed synaptophysin-pHluorin (sypHy)-based live cell imaging experiments. Primary SynI KO hippocampal neurons were co-transduced at 10 DIV with lentiviral vectors encoding sypHy and either SynI^WT^ or SynI^E373K^ fused to mCherry and analyzed 8–11 days after transduction (Fig. 9A).

We first evaluated the RRP, which in synaptic imaging accounts for both synchronous and asynchronous components of releasable SVs, by stimulating neurons with 20 APs @100 Hz [4]. While no genotype-dependent differences were present in the peak fluorescence, a significant increase in the time constant of fluorescence decay was observed in SynI^E373K^ as compared to SynI^WT^ terminals, revealing an impaired SV endocytosis, suggestive of defective recovery after RRP depletion (Fig. 9B). The lack of change in the RRP measured by sypHy imaging suggests that the decreased RRP_syn_ observed by patch-clamp recordings (see Fig. 7B) is accompanied by an increase of asynchronous release that may be facilitated by the increased resting Ca^2+^ (see Fig. 4). Next, to measure recovery after sustained stimulation, we performed repetitive RRP stimulations (20 APs @ 100 Hz) before or after a long-lasting stimulation (600 APs @ 20 Hz; Fig. 9C). Consecutive RRP stimuli resulted in a mild and not significant rundown in both SynI^WT^ and SynI^E373K^ synapses. Despite the peak fluorescence evoked by the first round of stimulation was not changed between the two genotypes, the RRP after the long-lasting stimulation was significantly reduced in SynI^E373K^ synapses (Fig. 9C). These data confirmed the defective SV recovery after sustained stimulation in SynI^E373K^ neurons.

### Ultrastructural analysis reveals that SynI^E373K^ affects SV trafficking at rest and during activity

To verify if the defects in the excitatory RRP_syn_ and recovery from synaptic depression in both excitatory and inhibitory synapses were due to alterations in SV trafficking, we evaluated the density and distribution of SVs in presynaptic terminals by electron microscopy of “asymmetric” (i.e., excitatory) and “symmetric” (i.e., inhibitory) synapses expressing either SynI^WT^ or SynI^E373K^.

**Fig. 10:**
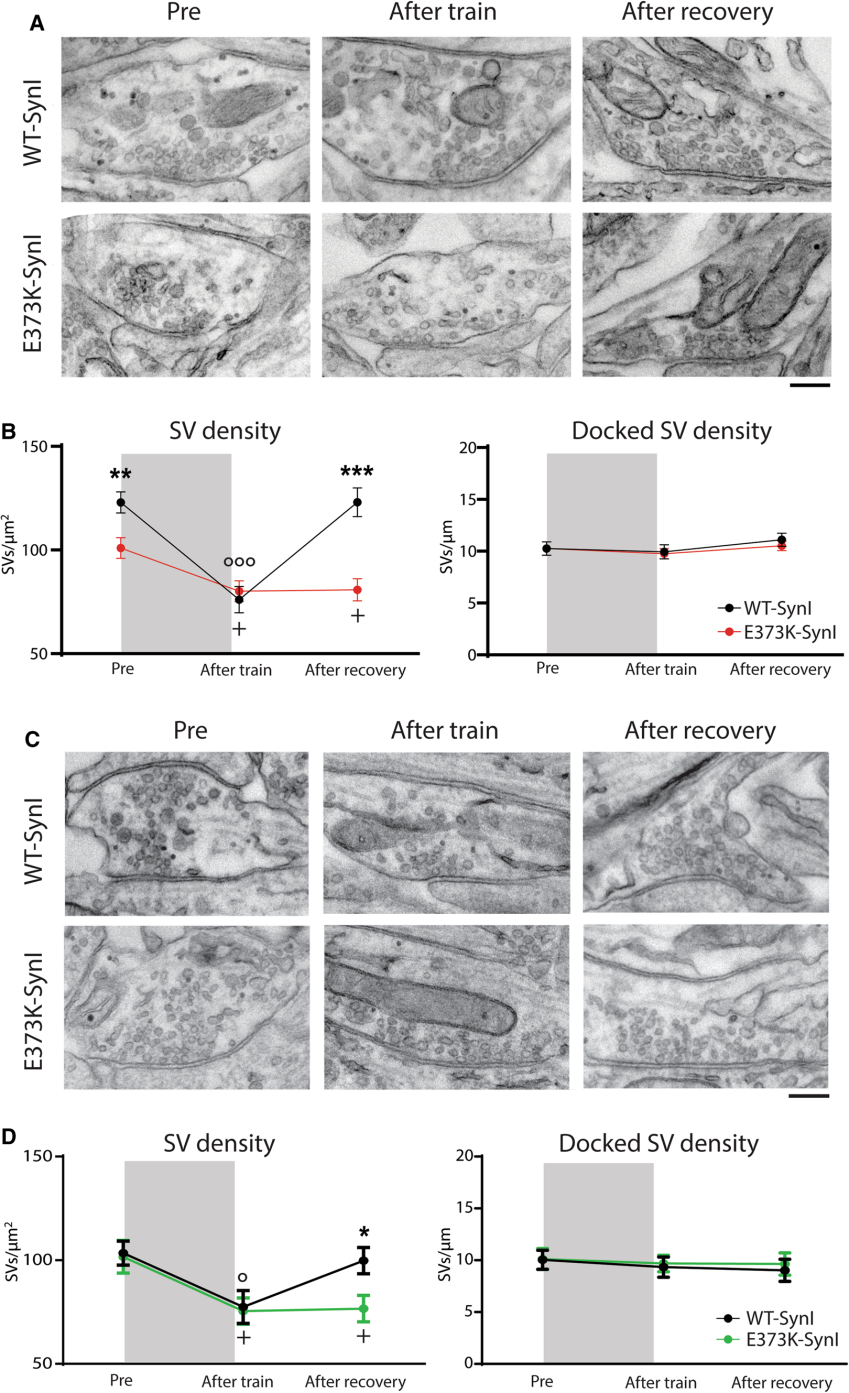
SV density and distribution are altered in excitatory and inhibitory synapses expressing the SynI^E373K^ mutant. **A, B** Excitatory synapses. **A** Representative transmission electron micrographs of excitatory presynaptic terminals from SynI KO neurons transduced with either SynI^WT^ (top) or SynI^E373K^ (bottom) and rapidly (1 s) fixed under resting conditions (*Pre*), immediately after a 30 s tetanic stimulation @ 20 Hz (*After train*) and following 120 s of recovery (*After recovery*). Scale bar, 200 nm. **B** Morphometric analysis of excitatory presynaptic terminals. For each genotype and experimental condition, the total SV density (*left*) and the number of docked SVs (*right*) are shown as means ± SEM. **C**, **D** Inhibitory synapses. **C** Representative transmission electron micrographs of inhibitory presynaptic terminals quickly fixed under the conditions as described in **A**. Scale bar, 200 nm. **D** Morphometric analysis of inhibitory presynaptic terminals. The total SV density (*left*) and the number of docked SVs (*right*) are shown for each genotype and experimental condition as means ± SEM. Excitatory and inhibitory synapses were manually identified based on their asymmetric or symmetric shape. Excitatory synapses: *n* = 30 and 30; inhibitory synapses: *n* = 15 and 15; for SynI^WT^ and SynI^E373K^, respectively, from 2 independent neuronal preparations. Across genotype: *p < 0.05; **p < 0.01; ***p < 0.001; unpaired Student’s *t*-test. Within genotype: °*p* < 0.05; °°°*p* < 0.001; *vs* “*Pre*” for SynI^WT^; ^+^*p* < 0.05 *vs* “*Pre*” for SynI^E373K^; one-way ANOVA/Dunnett’s multiple comparison test

Transduced excitatory and inhibitory synapses were analyzed at rest (1 s before the stimulation train) and at the following HFS (30 s @ 20 Hz) by quickly fixing the samples at the end of train and during the recovery period (2 min after the train) (Fig. 10A, C). At rest, excitatory SynI^E373K^ synapses showed a significant reduction in total SV density with respect to control SynI^WT^ synapses (Fig. 10B; *left panel*). Although no genotype-dependent changes were detected in the density of physically docked SVs (Fig. 10B; *right panel*), the decreased SV content is consistent with an impaired RRP refilling in mutant nerve terminals that could account for the functionally determined decrease in RRP_syn_ (see Fig. 7B). The total SV density was decreased under the condition of deep synaptic depression reached at the end of the stimulation train, but with no apparent genotype-dependent differences. However, at the end of the recovery period, excitatory synapses failed to recover the basal SV density (Fig. 10B; *left panel*), recapitulating the defective recovery evaluated electrophysiologically (see Fig. 8A). At rest and the end of the stimulation train, inhibitory SynI^E373K^ synapses were closely similar to control SynI^WT^ synapses in terms of total and docked SV densities (Fig. 10D). However, similarly to excitatory synapses, the total SV density of mutant inhibitory terminals failed to recover the basal SV density, consistent with the electrophysiological data (Fig. 10D; *left panel*).

Taken together, these results show that the absence of Ca^2+^ binding to SynI mainly affects excitatory synapses that fail to maintain a correct distribution of SVs under basal conditions. Moreover, in both excitatory and inhibitory synapses, the deletion of the Ca^2+^ binding site dysregulates the SynI-mediated trafficking of SVs in terminals challenged with sustained HFS.

## Discussion

Genetic analyses in human populations have identified several mutations in the *SYN1* and *SYN2* genes related to epilepsy and autism spectrum disorder [1, 17, 27, 31, 60, 65, 84], for a review, see [38]. Moreover, several studies of single and multiple Syn KO mice models confirmed the relationship between the *Syn1/Syn2* genes, epilepsy, and autism [16, 26, 35, 48, 55, 70]. Thus, the study of the precise functional role of Syns in excitatory and inhibitory synapses and their structure–function relationships within nerve terminals is fundamental to understanding the pathogenesis of such diseases.

SynI is present in nerve terminals at an average concentration of about 10–20 µM. It represents the 4% of SV proteins and 8–10 copies of SynI are associated with a single SV [20, 72, 79]. The crystal structure of the highly conserved central C-domain of SynI revealed a binding site for ATP, formed by a flexible MFL loop (residues 330–343), with high similarity with “ATP-grasp” enzymes [23]. In the proximity of the ATP binding site, SynI binds Ca^2+^ by coordinating it with the pyrophosphate moiety of ATP and two glutamate residues (Glu373 and Glu386). Structural studies and biochemical data on recombinant Syns suggested the possibility that ATP binding is regulated by Ca^2+^ [39]. Notwithstanding these data, the role of ATP binding to SynI and SynII has remained elusive. Based on the crystal structure of domain C, an increase in SynI tetramer formation upon binding of ATP in the presence of Ca^2+^ was reported [10]. More recently, using MD simulations and biochemical assays, we found that the MFL loop has a similarly reduced motility to holo-SynI also in the absence of Ca^2+^ and, consistently, SynI can bind ATP also in the absence of Ca^2+^ [62]. We also found that ATP binding strengthens the SynI association with SVs, favors the formation of high-order dimers and tetramers, and increases SV clustering. Under these conditions, the presence of Ca^2+^ negatively modulates SV clustering and favors the formation of tetramers over that of dimers [62] consistent with the previous structural predictions [10].

Deletion of ATP binding in SynI induced an increase in the probability of release at inhibitory synapses that potentiated the responses to single stimulation and increased synaptic depression [62]. Deletion of the same site in SynII also increased the probability of release and synaptic depression at excitatory synapses [75]. Although the two studies were done in distinct types of synapses, they show a similar functional outcome of the ATP binding site deletion in both Syn isoforms irrespective of the presence of Ca^2+^ binding, indicating the existence of distinct roles for Ca^2+^ and ATP binding to SynI/SynII. Since the micromolar ATP concentrations in nerve terminals do not represent a limiting factor for protein binding [67], it has so far been difficult to investigate separately the role of ATP binding to SynI from that of Ca^2+^ binding, and no specific role for Ca^2+^ binding to SynI has been proposed, except for facilitating ATP binding. Here, we show that ATP and Ca^2+^ binding by the SynI C-domain are independent events that, however, cooperatively potentiate each other. Thus, Ca^2+^ enhances ATP binding and, conversely, ATP increases the strength of Ca^2+^binding to SynI. To dissect out the physiological role of Ca^2+^ binding to SynI, we analyzed the effects of the E373K mutation that deletes the major Ca^2+^ binding site in SynI and induces a destabilization of the SynI oligomerization interface that is not dissimilar to that observed after deletion of the ATP binding site [62].

We found that the ablation of the Ca^2+^ binding in SynI had more severe consequences in excitatory than in inhibitory synapses. In the former synapses, the deletion of SynI-Ca^2+^ binding increased the frequency of mEPSCs without altering synaptic density and decreased the EPSC amplitude evoked by single stimuli. Distinct, but related, mechanisms are involved in these two effects. Under physiological conditions, SynI may act as a Ca^2+^ buffer, regulating the concentration of this ion in the presynaptic compartment. In the absence of Ca^2+^ sequestration by SynI, the resting Ca^2+^ concentration in excitatory nerve terminals could increase sufficiently to enhance spontaneous SV exocytosis that generates miniature events. Indeed, although the spontaneous release is independent of action potentials, it is at least partly Ca^2+^-dependent [44, 45, 59, 77]. This hypothesis is further confirmed by the observation that supplementation of an exogenous Ca^2+^ buffer completely occluded the effect of the SynI mutant. Why a similar effect is not occurring in inhibitory synapses that also express, to a large extent, SynI [9]? One possible explanation is that inhibitory neurons express a variety of Ca^2+^-buffering proteins [74], such as parvalbumin, calbindin, and calretinin [3, 22, 37, 41, 46, 77, 81] that characterize their peculiar functional properties. Under these conditions, the expression of Ca^2+^-buffering proteins by distinct classes of interneurons may render the contribution of the SynI-mediated buffering dispensable under resting conditions. It will be interesting to investigate whether the expression of parvalbumin in excitatory neurons occludes the effect of SynI^E373K^ mutation or, alternatively, whether silencing endogenous Ca^2+^ buffers in inhibitory neurons makes them sensitive to the E373K mutation.

On the other hand, the decreased EPSC amplitude due to a selective decrease in RRP size can be a consequence of the decreased SV density observed by electron microscopy in excitatory synapses under resting conditions. Although we did not detect any change in the density of physically docked SVs, the decrease in RRP can be explained by a decreased refilling with primed SVs from a more dispersed RecP. Such dispersion can be a consequence of: (i) the increased resting intraterminal Ca^2+^ that stimulates CaM kinases to phosphorylate SynI, favoring the release of SVs from the clusters and the actin cytoskeleton; and/or (ii) a possible mutation-induced change in SynI oligomerization, as suggested by MD simulations, that may alter the SV clustering capacity. Recent work demonstrated the capability of Syns to trigger liquid phase separation that participates in the clustering and maintenance of the SV pools in nerve terminals [57, 58, 64] and that is released upon Syn phosphorylation by CaM kinases [58]. Thus, a tonic activation of CaM kinases due to the increased concentration of resting Ca^2+^ could also impair SynI liquid phase separation and lead to SV cluster dispersion. While the deletion of the Ca^2+^ binding site in SynI can be defined as a loss-of-function, some of the phenotypes of SynI^E373K^ appear as gain-of-function when compared to SynI^WT^ and SynI^KO^. Indeed, SynI^E373K^ induces an increase in mEPSC frequency, as the consequence of the increase in resting free Ca^2+^, while constitutive depletion of SynI does not affect mEPSC frequency or amplitude [14]. On the other hand, constitutive deletion of SynI increases eEPSC amplitude and RRP size as a secondary effect of the impairment in inhibitory transmission [14, 25, 82], while SynI^E373K^ behaves similarly to SynI^WT^, indicating that the RRP size does not depend on Ca^2+^ binding to SynI.

Another effect of the deletion of Ca^2+^-binding to SynI, shared by excitatory and inhibitory synapses, consists of a sharp impairment in recovering PSCs after prolonged HFS. This effect finds its ultrastructural counterpart in a marked depletion of SVs at the end of the recovery period, indicating a defective RRP refilling. This effect, together with the impaired and slowed down retrieval of exocytosed SVs, suggests a modulation exerted by Ca^2+^ binding on the kinetics of endocytosis that could be mediated by the previously reported interactions of SynI with endocytic proteins such as intersectin and amphiphysin [24, 32, 61]. While all the remaining effects of the Syn mutant were exclusively found in facilitating excitatory synapses, the effects on recovery from depression also extended to an inhibitory transmission that is more prone to experience depression given its higher probability of release.

**Fig. 11:**
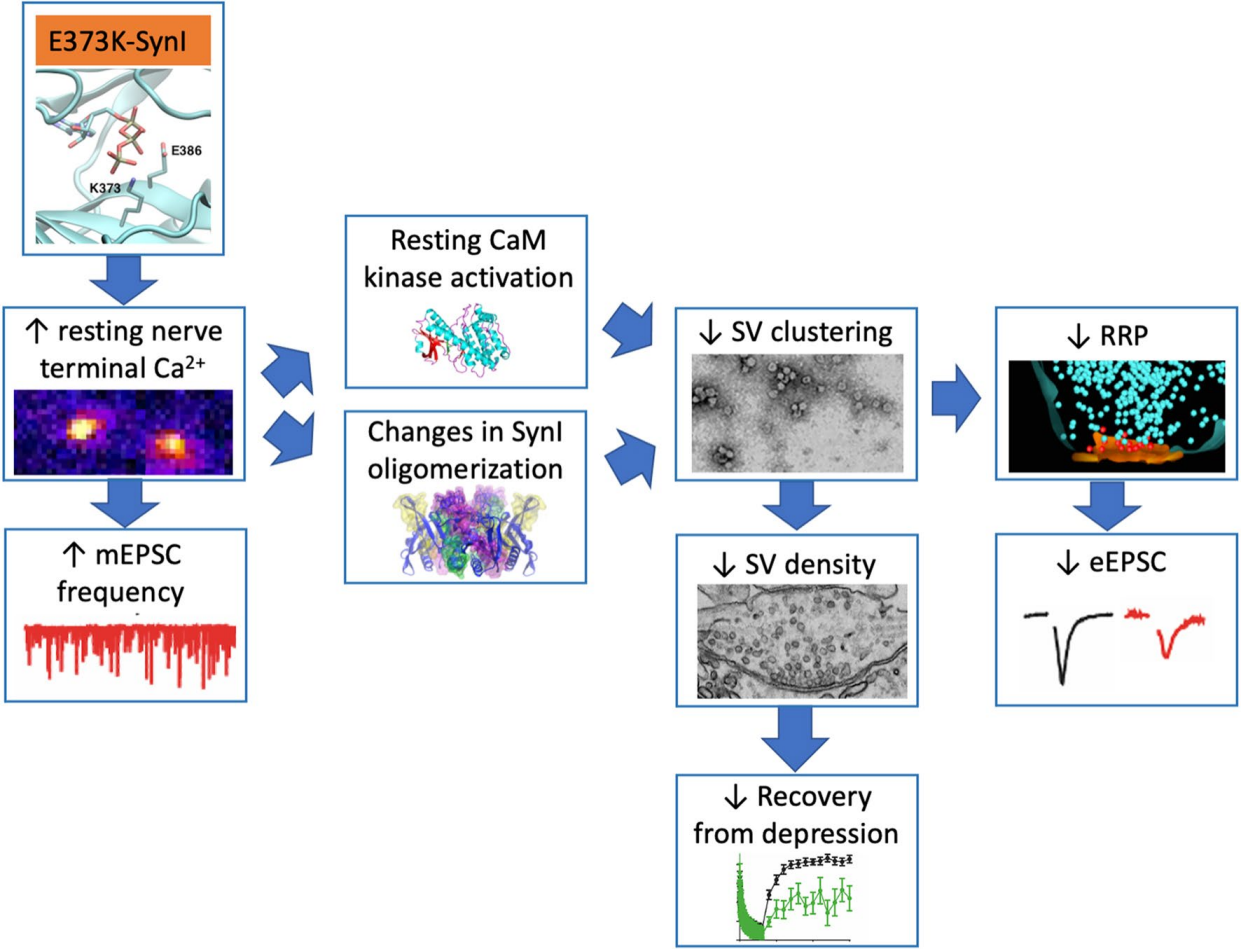
Schematic model of the functional consequences of the lack of Ca^2+^-binding to SynI in excitatory synapses. The lack of Ca^2+^-buffering by SynI in excitatory synapses, characterized by a low Ca^2+^-buffering capacity, results in an increased intraterminal [Ca^2+^]_i_ under resting conditions. This increases the probability of spontaneous SV fusion events and decreases the clustering of SVs in the RecP. This results in a decrease in RRP size for synchronous release and an impairment of the reconstitution of SV stores during recovery after HFS. The decrease in SV clustering may be contributed by an increased tetramerization of SynI and/or to increased phosphorylation of SynI by CaM kinases induced by the rise of resting intraterminal [Ca^2+^]_i_. Inhibitory synapses, endowed with a more efficient Ca^2+^-buffering capacity, are only affected by the lack of Ca^2+^-buffering by SynI under the intense Ca^2+^ build-up that occurs during HFS

The putative events triggered by the deletion of Ca^2+^ binding to SynI in excitatory synapses are schematically represented in Fig. 11. Inability to bind Ca^2+^ increases the resting Ca^2+^ concentrations which, in turn, increases the frequency of mEPSCs and constitutively activates CaM kinases phosphorylating SynI at distinct sites and promoting its dissociation from SVs and actin. This will in turn reduce SV clustering and disperse SVs in the RecP. The impaired SV clustering may be also contributed by the effect of the mutation on SynI oligomerization [62]. The dispersion of SVs in the RecP can, in turn, be responsible for the decreased RRPsyn and eEPSC amplitude, possibly due to a decreased SV priming and/or refilling of RRP, and for the marked impairment of EPSC recovery after prolonged HFS.

For the time being, the E373K mutation has not been identified in the *SYN1* gene of patients suffering from autism spectrum disorders or epilepsy. The first nonsense mutation in *SYN1* to be reported (W356X; [31] also deletes the Ca^2+^ binding site; however, the expression of the truncated form of synapsin I was markedly impaired, due to both the strongly decreased translation by non-sense mediated RNA decay (NMD) and severe intracellular aggregation of the NMD-escaped protein [33].

In conclusion, we have shown that Ca^2+^ binding to SynI is fundamental for maintaining a physiological SV organization at rest and during/after HFS. Excitatory neurons seem to be more sensitive to the Ca^2+^-dependent activity of SynI, indicating a putative role of SynI as Ca^2+^ buffer in these terminals under resting conditions. These findings contribute to the clarification of the distinct roles of ATP and Ca^2+^ binding to the highly conserved central C-domain of Syns and underline the central role of the regulation of Ca^2+^ basal levels and transients in nerve terminals in driving SV trafficking, synaptic transmission, and plasticity.

## Data Availability

The data and material that support the findings of this study are available upon request to the corresponding authors.
